# Reflections on a Copenhagen–Minneapolis Axis in Bioorganic Chemistry

**DOI:** 10.3390/molecules29061317

**Published:** 2024-03-15

**Authors:** George Barany, Paul R. Hansen

**Affiliations:** 1 Department of Chemistry, University of Minnesota, Minneapolis, MN 55455, USA; 2 Department of Drug Design and Pharmacology, Faculty of Health and Medical Sciences, University of Copenhagen, DK-2100 Copenhagen, Denmark; prh@sund.ku.dk

**Keywords:** orthogonality in chemistry, solid-phase peptide synthesis (SPPS), dithiasuccinoyl (Dts) protection of amines, glycopeptide synthesis, poly(ethylene glycol) (PEG)-based supports

## Abstract

The international peptide community rejoiced when one of its most distinguished members, Morten Meldal of Denmark, shared the 2022 Nobel Prize in Chemistry. In fact, the regiospecific solid-phase “copper(I)-catalyzed 1,3-dipolar cycloaddition of terminal alkynes to azides” (CuACC) reaction—that formed the specific basis for Meldal’s recognition—was reported first at the 17^th^ American Peptide Symposium held in San Diego in June 2001. The present perspective outlines intertwining conceptual and experimental threads pursued concurrently in Copenhagen and Minneapolis, sometimes by the same individuals, that provided context for Meldal’s breakthrough discovery. Major topics covered include orthogonality in chemistry; the dithiasuccinoyl (Dts) protecting group for amino groups in α-amino acids, carbohydrates, and monomers for peptide nucleic acids (PNA); and poly(ethylene glycol) (PEG)-based solid supports such as PEG–PS, PEGA, and CLEAR [and variations inspired by them] for solid-phase peptide synthesis (SPPS), solid-phase organic synthesis (SPOS), and combinatorial chemistry that can support biological assays in aqueous media.

## 1. Introduction 

This special issue of *Molecules* celebrates the scientific achievements of Morten Meldal of Denmark [1], who deservedly shared the 2022 Nobel Prize in Chemistry [2] with the Americans Carolyn Bertozzi and Barry Sharpless for “development of click chemistry and bioorthogonal chemistry”. Meldal’s specific discovery that led to the call from Stockholm was the so-called “copper(I)-catalyzed 1,3-dipolar cycloaddition of terminal alkynes to azides” (CuACC) reaction, first reported [3] at the 17^th^ American Peptide Symposium held in San Diego in June 2001, and amplified in a 2002 *J. Org. Chem*. citation classic [4]. However, the case is readily made that this breakthrough result was but one aspect of an ambitious, multifaceted, and highly successful research program [5] centered on the solid-phase synthesis of peptides, glycopeptides, and small organic molecules, along with applications of these advances to combinatorial chemistry. 

One of the authors (GB) of this personalized perspective^1^ carried out doctoral and postdoctoral studies for nine years (1971–1980) at The Rockefeller University in New York City with Bruce Merrifield, who would later become the 1984 Chemistry Nobel laureate [6,7,8] for “development of methodology for chemical synthesis on a solid matrix”. GB’s April 1977 Ph.D. thesis, reflected by a communication to the *J. Am. Chem. Soc.* later the same year [9], was the first time the term “*orthogonal*” was used in a chemistry context^2^^,^^3^. The concept was elaborated on further in a 284-page review chapter, with over a thousand references, on solid-phase peptide synthesis (SPPS) that was published in 1979 [12], but it remained until 1985 for the first experimental demonstration—reported from the Department of Chemistry at the University of Minnesota in Minneapolis in collaboration with Fernando Albericio of the University of Barcelona—of a three-dimensional orthogonal scheme for solid-phase peptide synthesis [13]. 

In the Fall of 1980, at the very start of his independent academic career in Minnesota, GB was summoned, on very short notice, to a meeting of untenured faculty members^4^ with Melvin Calvin, the 1961 Chemistry Nobel laureate [14,15] for discovery of the eponymous cycle that explains how carbon dioxide is assimilated in plants. Calvin, who coincidentally was then (and to this date remains) the only Ph.D. alumnus from Minnesota Chemistry so honored, happened to be in town and had made time to visit the alma mater. As each participant in the meeting was quizzed about their interests, GB self-identified as a bioorganic chemist. Calvin asked “What do you mean by that?” GB replied “…our goal is to use the traditional tools of organic chemistry, i.e., structure determination, total synthesis, and mechanistic studies, to solve problems of biological interest”. Calvin then uttered this unforgettable riposte: “Well, when I *invented* the term, ‘bioorganic chemistry’ was whatever I was interested in!”

The other author (PRH) of this article has conducted his entire educational and professional career in Denmark, save for an 18-month period (1993–1994) when he was a visiting Ph.D. candidate in the Barany laboratory at the University of Minnesota. PRH completed his doctoral studies under the supervision of Arne Holm, originally a classical organic chemist who, after several months in Merrifield’s lab at The Rockefeller University in the mid-1980s, pivoted his research so as to became a significant contributor to the peptide field through most of the 1990s [16,17,18,19,20]. This included co-invention of polystyrene-grafted polyethylene films with fellow Dane Rolf Berg, along with James Tam and R. B. Merrifield [21], and some collaborative projects with Morten Meldal [22,23]. In 1996, PRH earned his Ph.D. with a thesis, entitled “New Strategies in the Synthesis of 2-Acetamido-2-deoxy-β-d-glucopyranose *O*-Glycopeptides, Neoglycoconjugates and Photoactivatable Peptides”, that covered projects both in Minneapolis and in Copenhagen.

For his independent career on the faculty of the Department of Drug Design and Pharmacology at the University of Copenhagen, PRH’s research has covered the design, synthesis, and testing of antimicrobial peptidomimetics [24,25,26,27,28], along with peptides that can serve as probes in immunology [29,30]. During the past decade, PRH has collaborated with his Copenhagen colleagues Henrik Franzyk^5^, Anders Løbner-Olesen and Gunnar Houen. He adds the Danish perspective to the present contribution, and has evaluated the current legacy of the title “Copenhagen–Minneapolis axis”.

## 2. Motivations

It bears re-emphasis that our original forays into the various avenues of chemistry research reviewed herein were grounded in a strong desire to make meaningful contributions to biochemistry. In 1971, Bernd Gutte and R. B. Merrifield had just published their landmark *full* paper [34] on the total synthesis of an enzyme, ribonuclease A, by the solid-phase method [35]. It thus appeared to an enthusiastic neophyte^6^ that this accomplishment would open unlimited doors to understanding how proteins work and to engineering new proteins with tailor-made structures and specificities, among many other possible applications. However, for reasons that are outlined in the following, this turned into a “dream deferred” [37], as much further painstaking yet essential work was required for the remainder of the twentieth century, before at least some of those initial promises could be redeemed.

A deeper understanding of factors contributing to the success of the ribonuclease synthesis focuses on the chemical choices that are made about how to protect the various functional groups in the amino acid building blocks, along with creation of the essential linkage to the solid support—in all, the so-called “protection scheme”—and the corresponding cleavage conditions. 

**Scheme 1:**
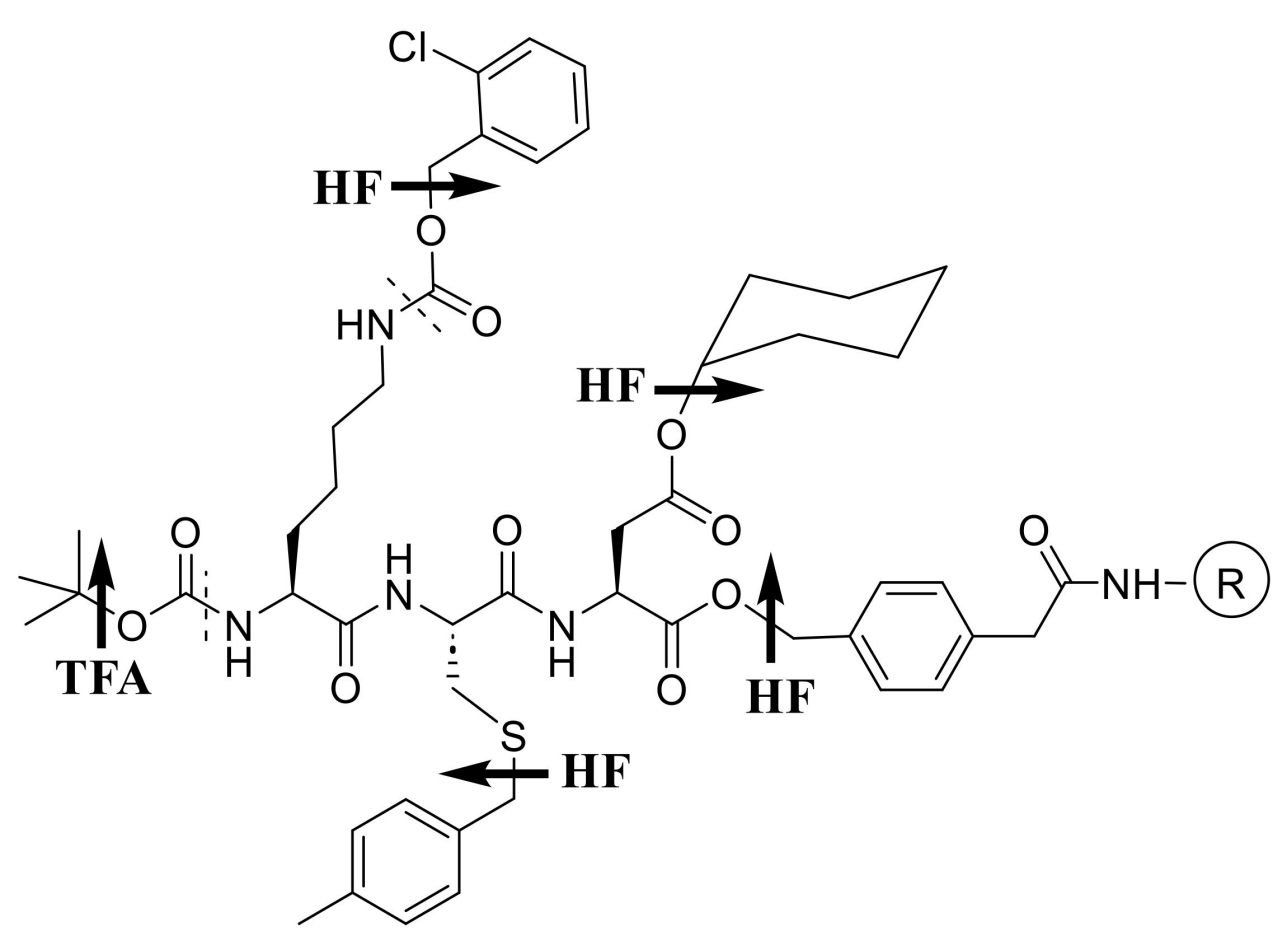
Optimized Merrifield protection scheme, exemplified with the hypothetical tripeptide Boc-Lys(ClZ)-Cys(Meb)-Asp(cHex)-PAM–resin. The same chemical mechanism, i.e., acidolysis, is used to remove both the “temporary” Boc group [38,39,40] and the “permanent” modified benzyl and cyclohexyl groups, as well as the phenylacetamidomethyl (PAM) linkage [41]. Therefore, a moderate-strength acid, e.g., trifluoroacetic acid (TFA), is used at each cycle for Boc removal [a *tert*-butyl carbocation undergoes E1 to produce isobutylene or S_N_1 to produce *tert*-butyl trifluoroacetate (tBuOTf), following which a carbamate intermediate spontaneously loses carbon dioxide (CO_2_) to provide the peptide trifluoroacetate salt, and a separate *neutralization* step with a tertiary amine, e.g., *N*,*N*-diisopropylethylamine (DIEA), is carried out before the next coupling]. For the final cleavage and deprotection, a strong anhydrous acid, e.g., anhydrous hydrogen fluoride (HF), is then used. Appropriate optimized protecting groups are known for the side-chains of all proteinogenic amino acids, and there are numerous options for the anchor, including some that give *C*-termini different from the standard *C*-terminal carboxyl [7,12,42].

Traditional stepwise SPPS chemistry (Scheme 1) relies on the acidolyzable *N*^α^-*tert*-butyloxycarbonyl (Boc) amino protecting group, which is removed at each cycle by anhydrous trifluoroacetic acid (TFA), or an acid of comparable strength. Side-chain protecting groups used, and the anchoring linkage, must then be sufficiently stable so as to withstand repeated TFA applications, yet be amenable to efficient and complete removal at the end of the synthesis by a stronger acid, typically anhydrous hydrogen fluoride (HF) in the presence of appropriate scavengers. The final acid strength is dictated by the requirements of a graduated lability scheme, yet numerous moieties of biological interest—including sugar, phosphate, and sulfate groups that represent post-translational modifications to peptides or proteins—cannot survive treatment with such acids. In addition, HF promotes a number of serious side reactions which have been well documented and can be mitigated to certain extents, but still have the potential to compromise yields and purities of peptides synthesized following this protection scheme.

**Scheme 2:**
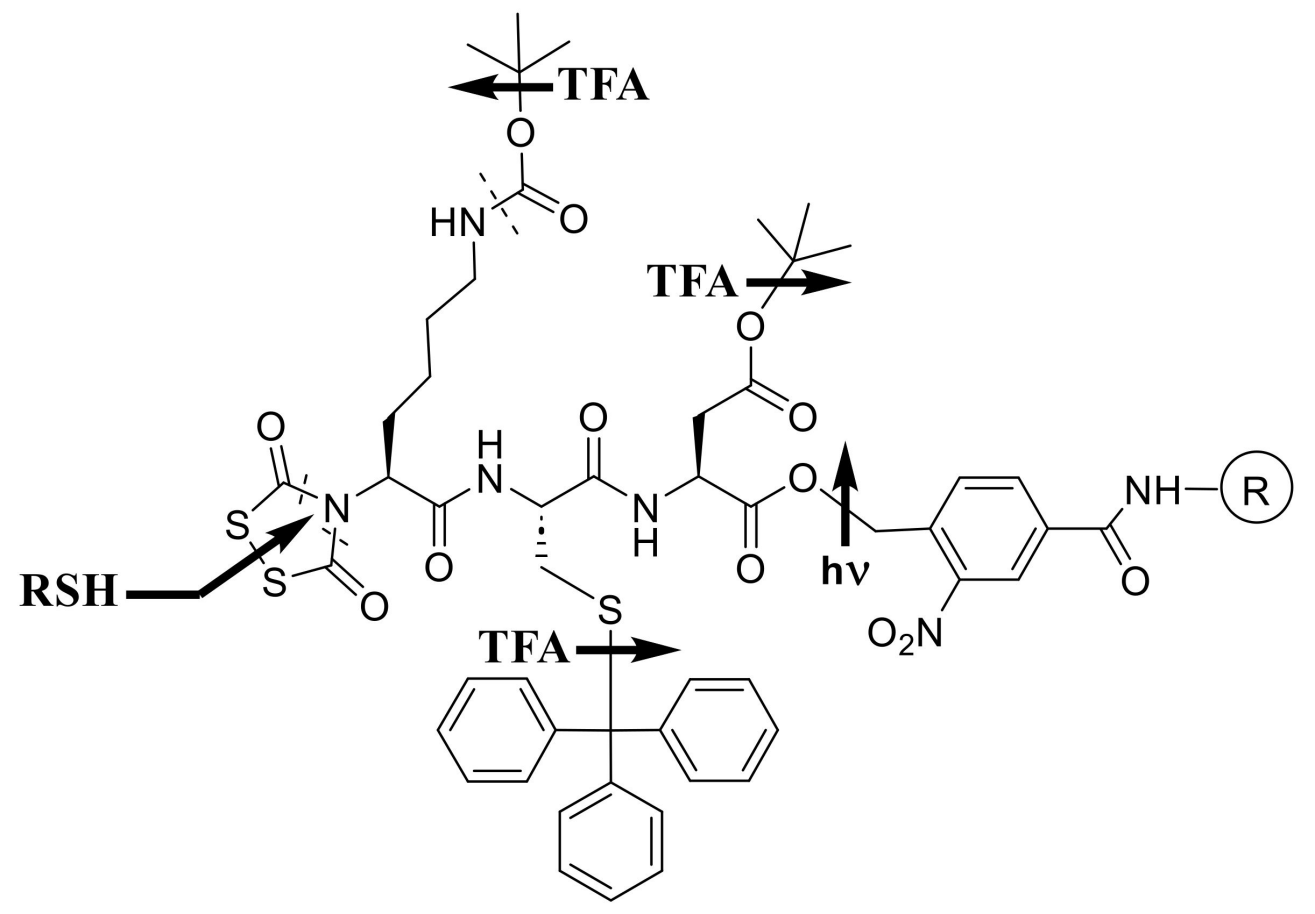
Three-dimensional orthogonal protection scheme [13], exemplified with the hypothetical tripeptide Dts-Lys(Boc)-Cys(Trt)-Asp(tBu)-ONb–resin. The “temporary” and “permanent” groups, and the anchoring linkage, can be cleaved, in any order, *via* three orthogonal mechanisms: (i) thiolysis or other reductive methods for dithiasuccinoyl (Dts) removal [9,13,44,45,46], wherein cleavage of the disulfide bond within the five-membered heterocycle is followed by loss of two molecules of carbonyl sulfide (COS); (ii) moderate-strength acid, i.e., trifluoroacetic acid (TFA), for cleavage of *tert*-butyl and trityl-based groups; and (iii) photolysis (350 nm) for cleavage of an *ortho*-nitrobenzyl ester [47] or amide [48].

We therefore envisioned an *orthogonal* alternative (Scheme 2) as a way to achieve *milder* overall conditions. Thus, Boc would be replaced by the acid-stable *N*^α^-dithiasuccinoyl (Dts) protecting group [9,13,43,44], removable by thiolysis or reduction, that we developed for just this purpose; side-chain protection would be “frame-shifted” mostly to acidolyzable *tert*-butyl (tBu) and triphenylmethyl (trityl ≡ Trt); and the peptide would be anchored *via* a photolyzable *ortho*-nitrobenzyl (ONb) ester. 

**Scheme 3:**
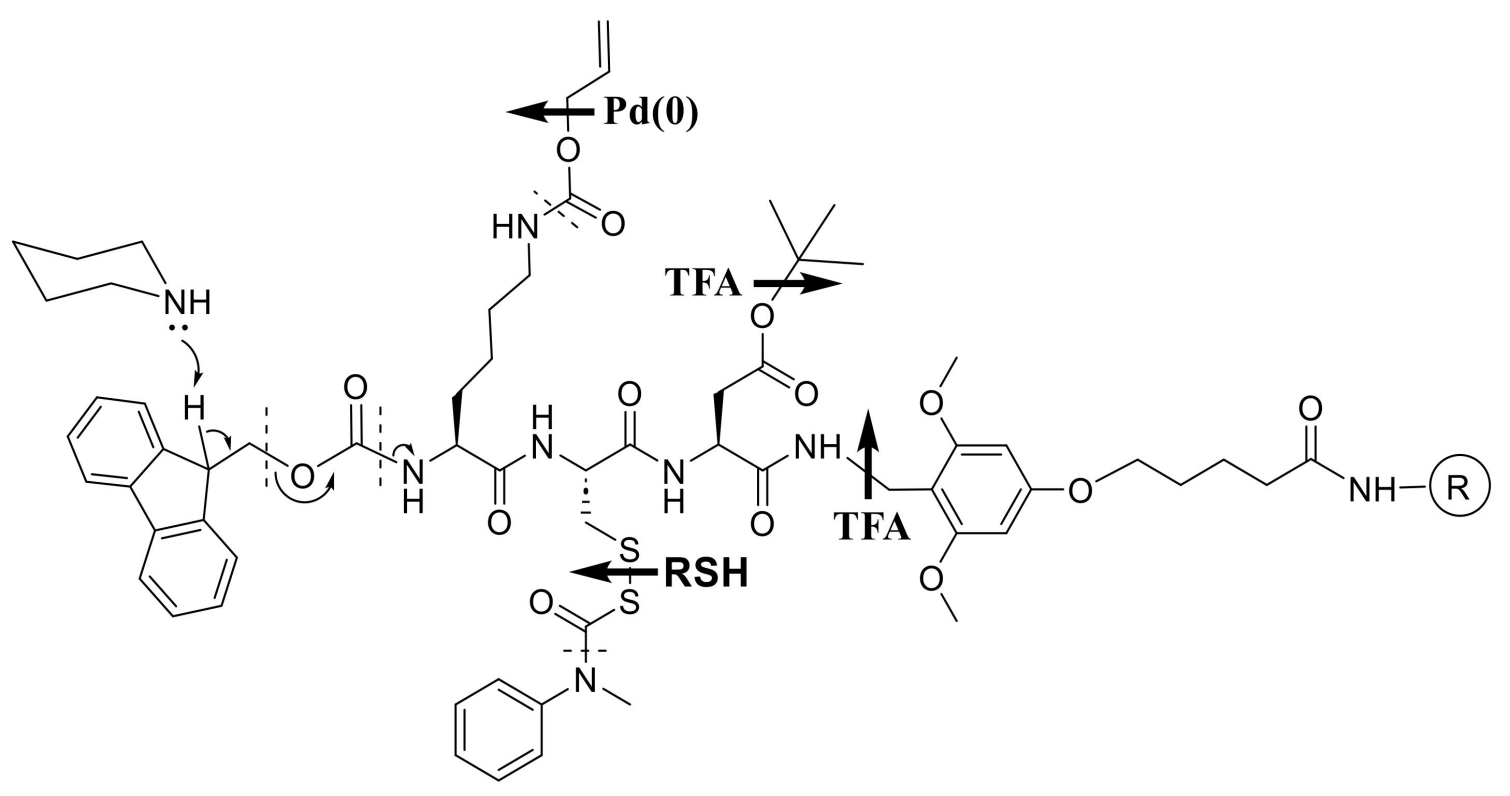
Orthogonal protection scheme, exemplified with the hypothetical tripeptide Fmoc-Lys(Aloc)-Cys(Snm)-Asp(tBu)-PAL–resin. Illustrated herein are additional concepts in peptide synthesis strategy, starting with the base-labile 9-fluorenylmethoxycarbonyl (Fmoc) “temporary” protecting group [50,51] that is removed by a β-elimination mechanism, with loss of carbon dioxide (CO_2_) from a carbamate intermediate. While most “permanent” protecting groups are then *tert*-butyl-based, with removal by acidolysis, several amino acid side-chains can be handled orthogonally, e.g., allyl-based protecting groups that are removed in the presence of palladium [52,53] and a thiolyzable (*N*-methyl-*N*-phenylcarbamoyl)sulfenyl (Snm) [54,55] group which can also be the electrofugal partner in directed disulfide formation carried out on-resin or in solution. The *S*-acetamidomethyl (Acm) group [56], which is removable by silver or mercuric salts [provides free thiol, after metal removal] or oxidizing agents such as iodine [leads to disulfides, both on-resin and in solution], also satisfies the criteria of orthogonality. Finally, this Scheme shows tris(alkoxy)benzylamide [**p**eptide **a**mide **l**inker ≡ PAL] anchoring [57,58], which results in creation of a *C*-terminal amide during the final acidolytic cleavage step. Relatedly, **b**ackbone **a**mide **l**inker (BAL) anchoring [59,60,61,62] provides even more flexibility in the protection scheme and allows entry to a myriad of linear and cyclic peptides.

In parallel to our work with Dts protection, additional orthogonal schemes emerged (Scheme 3) that are centered around the acid-stable, base-labile *N*^α^-9-fluorenylmethoxycarbonyl (Fmoc) group [49,50]. 

Chemistries exemplified by Scheme 1 and Scheme 3 became highly optimized during the 1980s and 1990s, as has been expertly reviewed elsewhere [51,63,64,65,66,67], while the scope and limitations of Dts chemistry (Scheme 2) have also become clear, as described in subsequent sections of the present article. Many of the goals that we had originally set for Dts actually came to fruition with Fmoc instead, and it was a privilege to contribute [57,58,68,69,70,71,72,73,74], along with many other investigators worldwide, to this grand agenda.

## 3. Orthogonal Chemistry

The concept of *orthogonality*, together with its numerous and varied experimental manifestations, has proven to be significant and influential in the development and maturation of the peptide synthesis field [75]. It has been generalized to cover not only protection schemes, but also a wide range of bond-forming reactions. When one of us (GB) first used the word while a graduate student making presentations at the Monday Merrifield group meetings at The Rockefeller University, he was repurposing something he had learned during his linear algebra classwork as a way to succinctly express something that was implicit in the work of leading chemists such as E. J. Corey [76]. 

In our framing, an orthogonal system was defined as a set of completely independent protecting groups in which different chemical mechanisms are used to remove each set (see footnote 2 in the introductory section of this perspective). During GB’s Ph.D. thesis defense in 1977, pushback came from the committee chair, 1972 Chemistry Nobel laureate Stanford Moore [77], who saw no advantage over the term “chemoselective”, while outside reviewer Ronald Breslow (Columbia University) [78] was likewise underwhelmed. 

Despite these tentative origins, “orthogonal” has eventually become embedded into the vocabulary^7^ of organic chemistry and related fields [79]. In the early 2000s, while Sharpless popularized the term “click chemistry” [80], Bertozzi [81] brought orthogonality to another level by adding the three-letter prefix “bio”—thereby championing whole families of highly specific coupling reactions [82,83,84,85,86] (variously referred to as “conjugations” or “ligations”) that can be carried out efficiently and selectively under the aqueous conditions that living systems function in^8^. 

## 4. Dithiasuccinoyl (Dts) Chemistry: Synthesis and Mechanisms

**Scheme 4:**
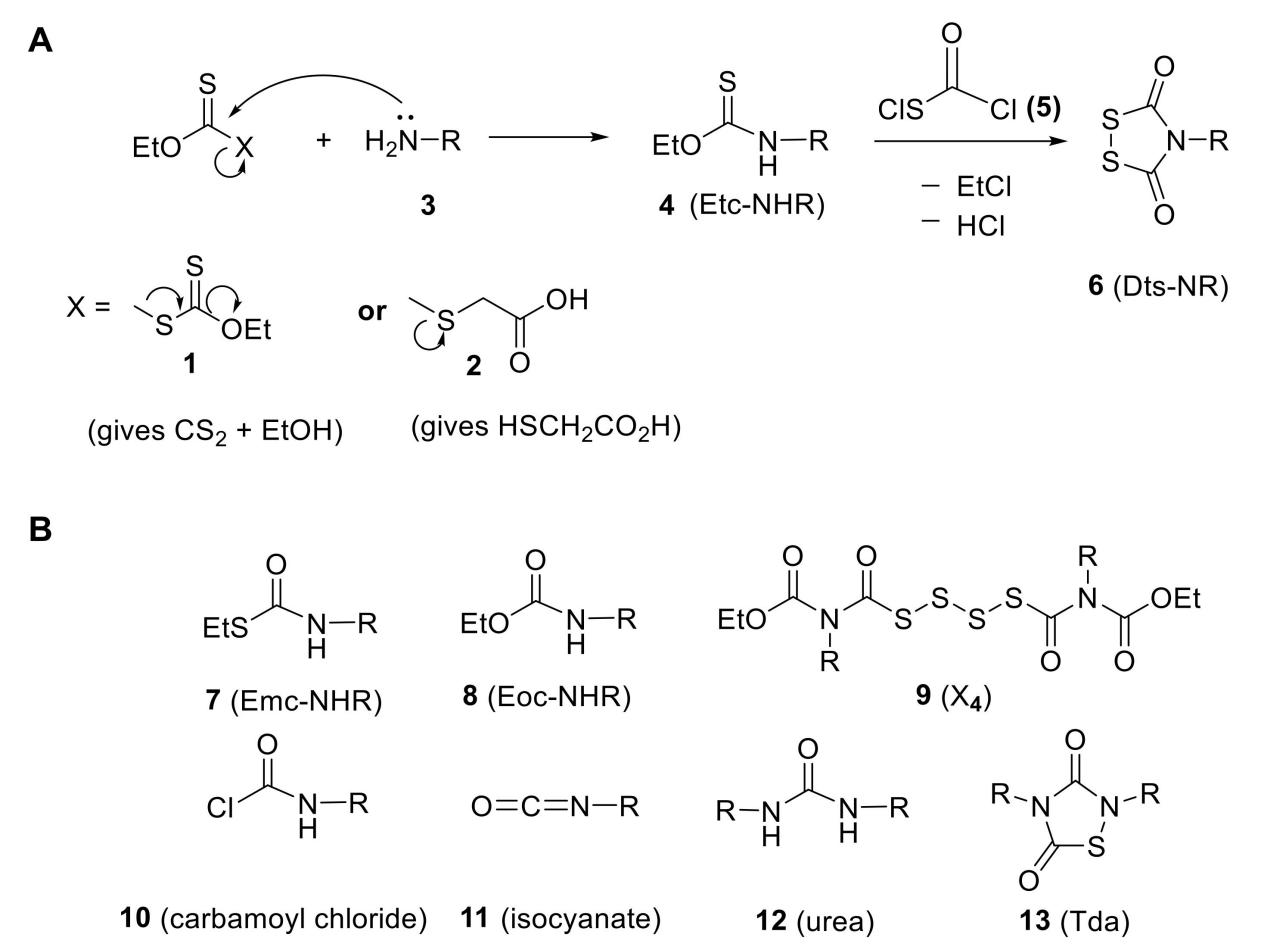
(**A**) Creation of *N*-ethoxythiocarbonyl (Etc) amines, followed by the Zumach–Weiss–Kühle (ZWK) reaction to introduce dithiasuccinoyl (Dts) protection to amines. All other things being equal, reagent **1** is preferred for derivatization of amino acids and reagent **2** is preferred for derivatization of aliphatic or aromatic amines that do not have other ionizable functional groups—in both cases, this is due to the ease of extractive workups which provide desired precursor **4** in excellent yields and exceptionally high purities [94]. The ZWK reaction that follows is optionally carried out in the presence of bases that serve as HCl acceptors, or on *N*-trimethylsilylated substrates where TMSCl is produced instead of HCl. This shown two-step sequence, and its variations, have been implemented successfully for a wide range of R groups. (**B**) Structures of compounds encountered as low-level, albeit yield-diminishing, by-products of the ZWK reaction. Of the three species retaining the Et group, **7** and **8** are described for several R groups in a few of our publications, while **9** (R = CH_3_) has to date only been mentioned at a few conferences [Barany, Słomczyńska, Mott, and Eastep]. The major species of **9**, as drawn, is referred to as X_4_; X because for quite a while it was an unknown structure, and the subscript 4 because it has four sulfurs (note that X_3_, X_5_, X_6_, and even higher polysulfanes are also observed). All four species that have lost the Et group, i.e., **10**–**13**, have been isolated or inferred in multiple systems, and been discussed in the primary refereed literature.

In the quest for an *N*^α^-amino protecting group that would be removed under mild orthogonal conditions, we realized that the 1,2,4-dithiazolidine-3,5-dione heterocycle (**6**)—described previously in the patent literature [89]—could be harnessed for this new purpose^9^. Starting from a parent amine **3**, elaboration of the heterocycle could be achieved in two facile steps (Scheme 4): first, conversion to an *O*-ethyl thiocarbamate (Etc) derivative (**4**), using either bis(ethoxythiocarbonyl)sulfide (**1**)^10^ or *O*-ethyl *S*-carboxymethyl dithiocarbonate (**2**) [94]; and second, applying the bifunctional (chlorocarbonyl)sulfenyl chloride (**5**) to effect what we call the “Zumach–Weiss–Kühle (ZWK) reaction”. 

Over a period of four decades, we have developed a thorough mechanistic understanding of the canonical ZWK reaction (Scheme 4A), including several variations to its original formulation^11^^,^^12^^,^^13^ [9,95,100,101,102]. A wide range of R groups (parent amine = H_2_N–R (**3**); note that the chemistry is different for R = H) have been studied, and conclusions were in some cases corroborated by sophisticated isotope labeling/mass spectrometric analytical experiments. However, for purposes of the present perspective, we would like to focus on two aspects: first, the pervasive presence of several low-level by-products from the ZWK reaction (compounds **7**–**13** in Scheme 4B) and second, the adjustments that were necessary in order to introduce the Dts group onto the α-nitrogen of amino acids in ways that would create derivatives of sufficient purity to be useful as building blocks for stepwise SPPS. 

Several of the by-products (Scheme 4B) to the ZWK reaction (and some of its variations)—namely carbamoyl chlorides (**10**), isocyanates (**11**), ureas (**12**), or novel 1,2,4-thiadiazolidine-3,5-diones (Tda’s) (**13**)—are readily explained as being due to decomposition of non-productive intermediates that form after the alkyl chloride (EtCl in Scheme 4A) has been expelled. Other than the annoyance that their formation leads to somewhat diminished overall yields, these species have considerably different physical properties from the desired Dts derivatives, and are removed without much difficulty upon standard extractive workups and/or straightforward recrystallizations.

Much more concerning are two types of urethane by-products that retain the alkyl group (i.e., ethyl (Et) in Scheme 4B). Formation of the Emc isomer **7** is not surprising, since the so-called “thionocarbamate—thiocarbamate rearrangement” is well precedented in organosulfur chemistry^14^. Creation of the Eoc urethane **8**, in which the sulfur of starting **4** is formally replaced by oxygen, has been shown to be due to the presence of small amounts of water under the nominally anhydrous reaction conditions of the ZWK reaction. This important conclusion was reinforced when water was added *intentionally* and the remarkable tetrasulfane **9** (along with a distribution of corresponding tri-, penta-, hexa-, and even higher polysulfane analogues) was observed, isolated, and proven by mass spectrometry and X-ray crystallography. Behind all of these fascinating mechanistic forays lay an uncomfortable truth: even low levels of by-products such as **7** or **8** would doom the application of Dts for peptide synthesis, because at every orthogonal Dts removal cycle, the residual urethane or thiourethane would remain unaltered and therefore terminate growing peptide chains. 

Our first breakthrough [100] concerning the preparation of ultra-pure Dts derivatives involved replacement of the ethyl group [in **4** (Etc-NHR)] by a 2-(dimethylamino)ethyl moiety [i.e., using (CH_3_)_2_NCH_2_CH_2_O(C=S)NH–R ≡ Dmaetc-NHR as the starting substrate for the ZWK reaction]. Now, all urethane-type by-products, along with the major co-product alkyl chloride, were simply removed upon aqueous extractive workup during the acid wash step. Starting with Dmaetc-NHR, instead of with Etc-NHR, had the further advantage of installing a “built-in” stoichiometric tertiary amine moiety to absorb the hydrogen chloride (HCl) that formed in the ZWK reaction.

**Scheme 5:**
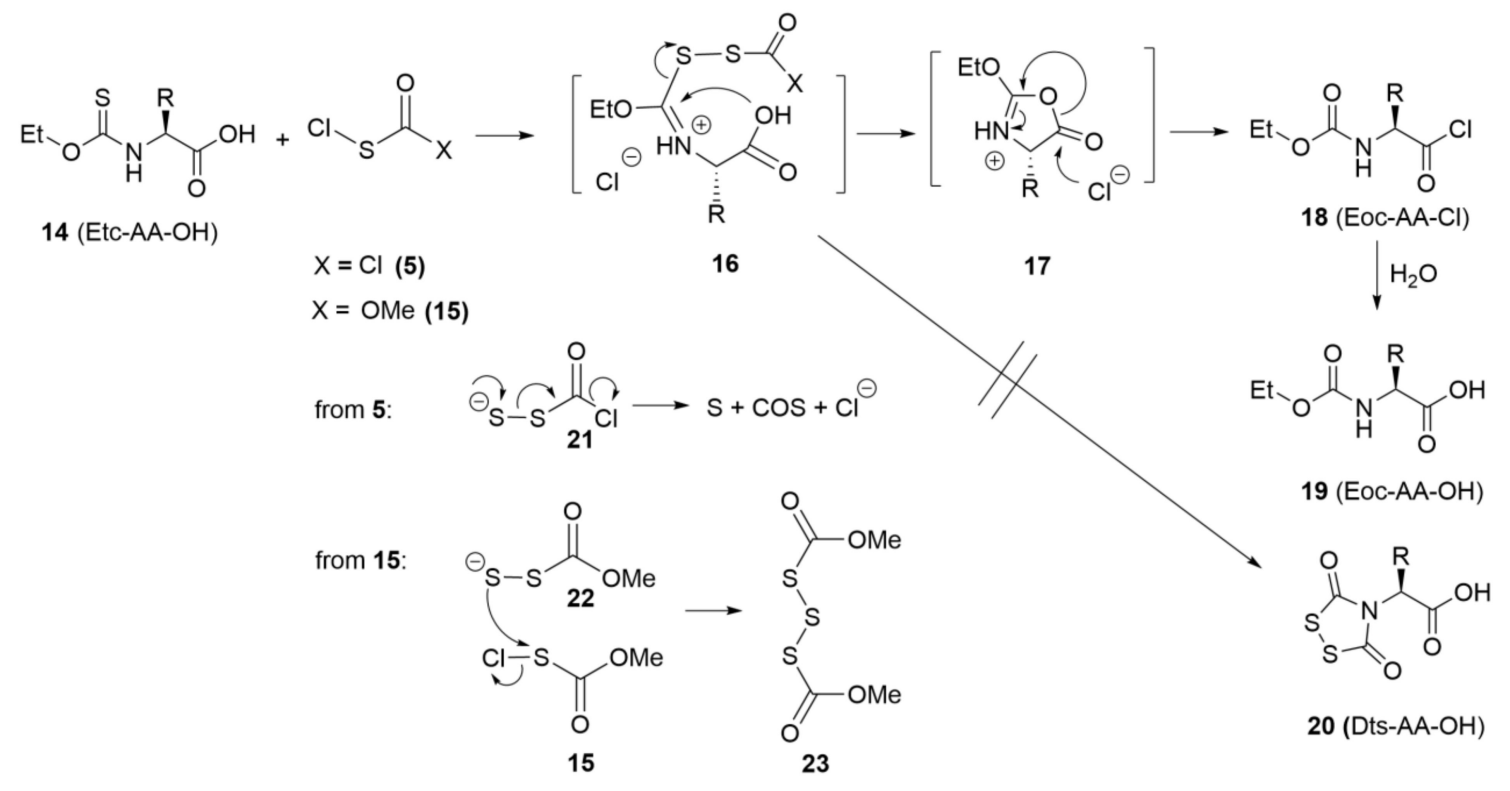
Attempts to prepare Dts-amino acids (**20**) by direct application of ZWK chemistry to Etc-amino acids (**14**) with free α-carboxyl groups [101]. Thionourethane substrates **14** were treated with sulfenyl chlorides **5** or (as a control) **15**. Depending on the precise conditions of the workup, urethane derivatives **18** (an acid chloride) or **19** (free α-carboxyl) were the exclusive amino acid-containing products. Co-products when using **5** were elemental sulfur (S), gaseous COS, and HCl (the acid, or its salt if the reaction was carried out in the presence of a tertiary amine). When **15** was used, the principal organic co-product was bis(methoxycarbonyl)trisulfane (**23**), admixed with smaller amounts of the corresponding di- and tetrasulfanes (formed *via* disproportionation); all structures confirmed by comparison to standards made by alternative, unambiguous methods [103]. This Scheme, by invoking intermediates **16** and **17**, shows likely mechanisms for *all* of these results. *Successful* methods to prepare Dts-amino acids (**20**) are described in the text.

In parallel to the various explorations summarized in the preceding paragraphs, we tried to obtain Dts-amino acids (**20**) needed to pursue our SPPS goals by treating the corresponding Etc-amino acid precursors (**14**), with free α-carboxyl groups, with (chlorocarbonyl)sulfenyl choride (**5**) according to optimal ZWK conditions (Scheme 4A). These new experiments were abject failures, but in keeping with one of GB’s life philosophies^15^, the full story proved to be fascinating and instructive (Scheme 5). 

Astonishingly, the major product was the ethoxycarbonyl (Eoc) urethane, albeit as the acid chloride (**18**), which could hydrolyze to the Eoc-amino acid (**19**) upon workup. This result was explained by intramolecular attack (five-membered transition state) of the free α-carboxyl group on the initial adduct **16**, displacing elemental sulfur (S), gaseous carbonyl sulfide (COS), and chloride. The five-membered ring intermediate **17** formed in this manner fragments further to provide the observed products (see Scheme 5 and its accompanying legend). 

How then to create Dts-amino acids? The ZWK reaction proceeds very smoothly when the substrate is an Etc-amino acid *ester*, typically an ethyl (OEt) or methyl (OMe) ester, and occasionally a *tert*-butyl (OtBu) ester. Normally, such esters are removed by simple saponification^16^, but that was not an option due to the base-lability of Dts^17^. It was then that we encountered our first dramatic validation of orthogonality for Dts chemistry, because it was possible to remove the ester by acid hydrolysis at elevated temperature [e.g., 40 h reflux in the presence of 0.15 m *p*-toluenesulfonic acid (TsOH) in HOAc–H_2_O (4:1)], while leaving the Dts group intact.

Obviously, one cannot rely on acid hydrolysis to create Dts derivatives of trifunctional amino acids, because the side-chain protecting groups that are likely to be relied on would certainly not survive those conditions. Fortunately, there was yet another tool in our kit: the α-carboxyl of an Etc-amino acid (**14**) can be easily and quantitatively converted *in situ* to the corresponding trimethylsilyl ester, using reagents such as *N*,*O*-bis(trimethylsilyl)acetamide (BSA) (**28**) or *N*,*N*′-bis(trimethylsilyl)urea (BSU) (**29**), in acetonitrile (preferred) or other aprotic solvents or solvent mixtures of similar polarity. The resultant Etc-AA-OTMS could then be transformed with (chlorocarbonyl)sulfenyl chloride (**5**) (ZWK reaction) to elaborate the Dts function, and then straightforward aqueous workup removed the TMS ester and provided the desired Dts-amino acids (compounds **20**, with the α-nitrogen protected and the α-carboxyl free). Yields were good, but the problem with low-level Eoc urethane by-products (**19**) persisted.

**Scheme 6:**
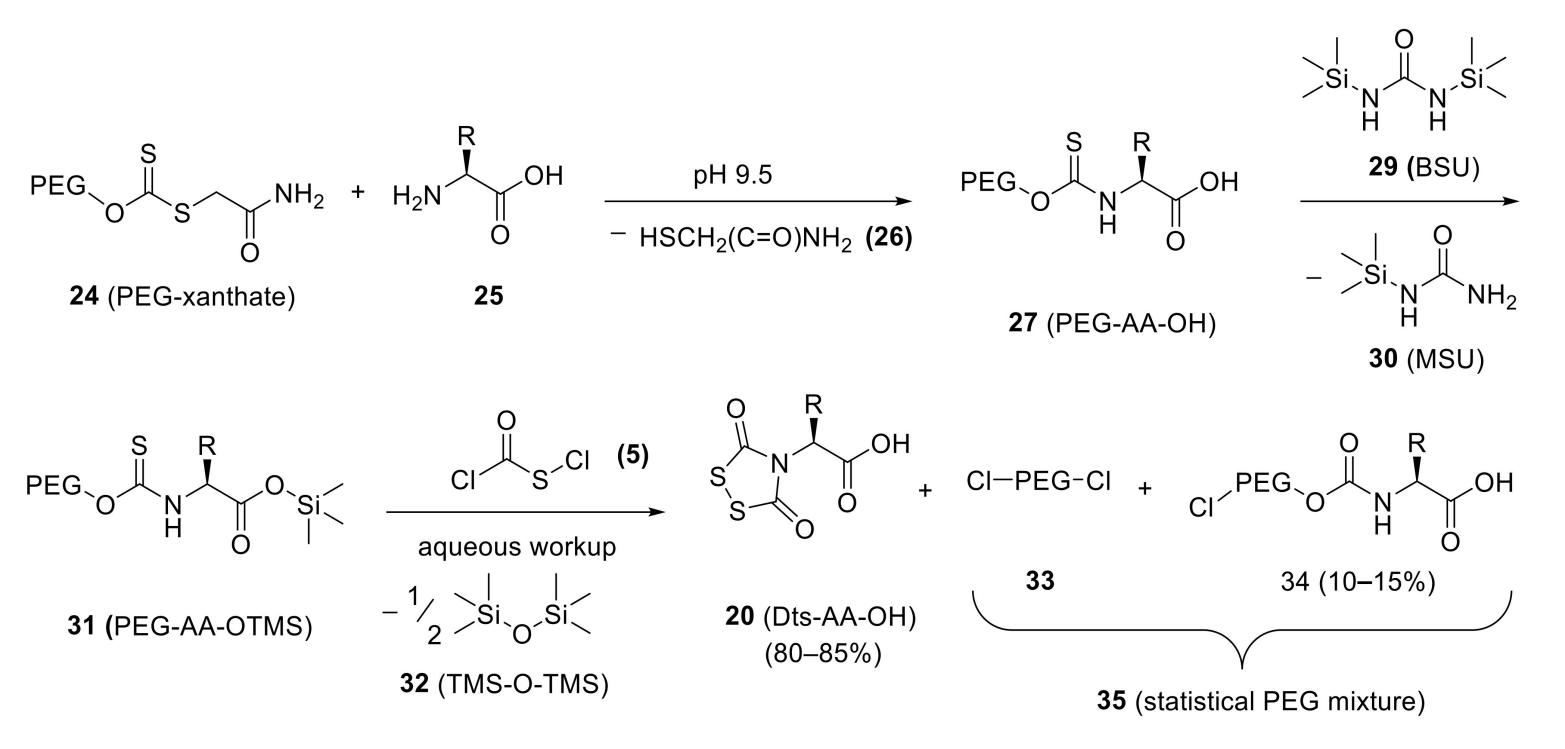
A robust and general PEG-based method for racemization-free synthesis of exceptionally pure Dts-amino acids (**20**), devoid of urethane-type by-products, as reported in detail from our Minneapolis laboratories in 1987 [101]. “One-pot” conversion of **27** to **20** works in a range of aprotic solvents, of which we found acetonitrile to be particularly convenient. The silylating agent to convert **27** to **31** *in situ* can be *N*,*O*-bis(trimethylsilyl)acetamide [BSA (**28**), drawn later, in Schemes 10 and 18] or *N*,*N*′-bis(trimethylsilyl)urea [BSU (**29**), as shown herein], which is slightly preferable. The overall method is viable for a range of PEG sizes, but our work was focused on a *bifunctional PEG*, HO(CH_2_ CH_2_O)_45_H, with an average molecular weight of 2000. For purposes of this Scheme, PEG derivatives **24**, **27**, and **31** are bifunctional, whereas both end groups are specified for PEG derivatives **33** and **34** [while **34** is stated to represent 10–15% of the polymers comprising the statistical mixture **35**, it should be recognized that the amount of amino acid present as the polymer-bound urethane is actually 5–8% because only one functional group of bifunctional PEG carries the amino acid]. Note that there is no need for a tertiary amine HCl acceptor in the ZWK reaction of **31** plus **5**, since the HCl formed either reacts with excess silylating reagent **28** or **29**, or acts to remove the trimethylsilyl ester of **31** directly.

The efforts to (i) understand the chemistry of the ZWK reaction and (ii) optimize yields and purities of Dts-amino acids merged once Samuel Zalipsky joined the Minneapolis team shortly after completing his M.Sc. work at the Hebrew University under the supervision of Chaim Gilon. GB suggested that Zalipsky leverage his expertise with chemical manipulations of poly(ethylene glycol) (PEG) in order to develop reagents for carrying out *polymer-bound* ZWK chemisty. We reasoned that by replacing the ethyl (Et) group (Scheme 4A) with PEG (Scheme 6), any urethane-type by-products (Scheme 4B) would have their solubility properties altered significantly enough to allow their easy separation—termed *functional purification*—from the desired Dts-amino acids (**20**, with free α-carboxyl) which are detached from the polymer. According to this plan, desired **20** can remain in organic solution, or be extracted into aqueous bicarbonate followed by acidification to return to an organic solvent, whereas any species attached to PEG could be extracted into water, or be precipitated out of organic solution by the addition of diethyl ether. 

In practice, the plan worked very well (Scheme 6). First, we prepared, on multi-kilogram scales, α,ω-bis[(carbamoylmethylthio)thiocarbonyl]oxypoly(oxyethylene) [structure **24** in Scheme 6, informally called “PEG-xanthate”]; this is a universal reagent that converts α-amino acids (with suitable side-chain protection, as needed) to the corresponding PEG-bound thionourethane starting points (**27**) for further transformations to elaborate the Dts heterocycle. Ultimately, we were able to prepare—in reasonably good yields and excellent purities—a complete set of Dts derivatives with appropriate side-chain protection for 19 of the twenty proteinogenic amino acids [101] (the twentieth being the α-imino acid proline, which can be protected by a suitably fine-tuned open-chain carbamoyl disulfide [101,107], as shown in structure **54**, later in this perspective). Dts-amino acid derivatives were crystalline, either directly or as dicyclohexylammonium (DCHA) salts^18^. 

Working with PEG derivatives had the added benefits that all intermediates were amenable to purification, either by “crystallization” from ethanol or by precipitation with cold diethyl ether, and could be characterized by standard methods of organic chemistry, including elemental analysis, ultraviolet (UV) absorption spectroscopy, and ^1^H as well as ^13^C nuclear magnetic resonance (NMR).

There was one further aspect of this enterprise that began as an academic exercise in scholarly rigor, but would prove to have an unexpected payoff, as will be covered later in this perspective. In order to definitively prove the formation of PEG-bound urethanes that would later be removed according to our functional purification concept, we needed to isolate and characterize heterobifunctional species **34** out of a statistical mixture (**35**) that was primarily PEG-Cl (**33**) [see Scheme 6 for structures of **33**, **34**, and **35**]. This objective was achieved by ion-exchange chromatography on DEAE-Sephadex A. 

The aforementioned PEG chemistry, with its applications for the preparation of Dts-amino acids, was spearheaded by Samuel Zalipsky, and significant experimental contributions were made by Fernando Albericio (previously introduced), Urszula Słomczyńska (chronologically, GB´s very first postdoctoral fellow, from Łódź in Poland), and Adele Binning (an undergraduate then, who later went on to be a curator at the Minnesota Science Museum). Large batches of PEG-xanthate (**24**) were made collaboratively, first with Steven Heilmann of the 3M company in Saint Paul, Minnesota, and later with Derek Hudson of Biosearch, then a biotechnology startup in San Rafael, California. 

## 5. Dithiasuccinoyl (Dts) Chemistry: Thiolytic Deprotection and Applications to SPPS

Reinforcing points made earlier in this perspective, we recognized in Dts-amines a number of structural characteristics that made these derivatives promising candidates to satisfy our goal to develop a new amino protecting group that could be removed under mild, orthogonal conditions. In biochemical systems, there are few reactions as facile as disulfide bond cleavage through reaction with a suitable thiol. Dts-amines incorporate a disulfide bond as part of a five-membered ring, and there is a great driving force for disulfide cleavage because it relieves the unfavorable strain of a 0° dihedral angle [note that for disulfides, the optimal dihedral is 90° so as to minimize repulsion between unshared p*π* electrons]—this results in a rate increase of *four orders of magnitude* by comparison to standard thiol-disulfide exchange reactions [43,108]. Moreover, the reaction is *not* an equilibrium, but is rather driven to completion by irreversible loss of two equivalents of carbonyl sulfide (COS)^19^.

**Scheme 7:**
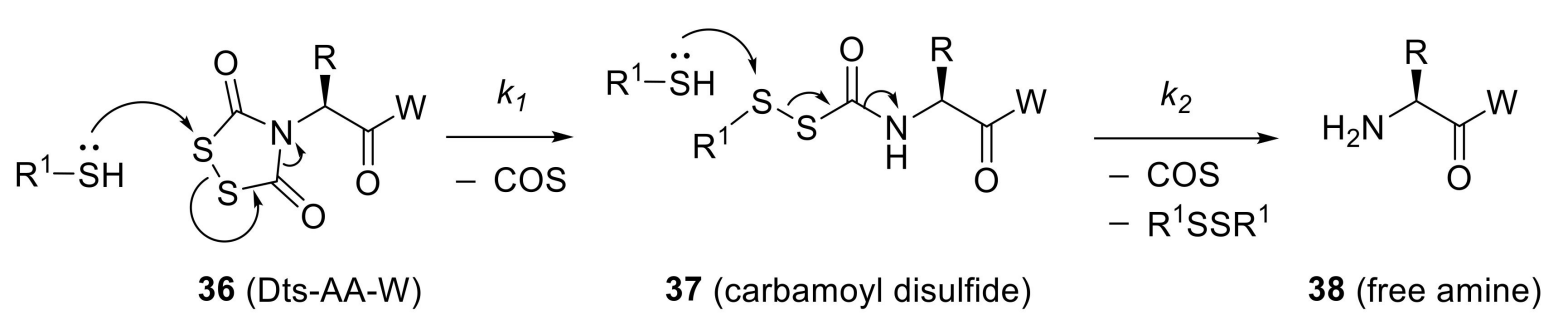
Thiolytic removal of the dithiasuccinoyl (Dts)-amino protecting group, *via* an open-chain carbamoyl disulfide intermediate, as established in a comprehensive kinetic/mechanistic study [43,46] carried out mostly on Dts-glycine (R = H; W = OH). The rate constants *k*_1_ and *k*_2_ were determined for a wide range of thiols (R^1^–SH), a variety of solvents, and in the presence or absence of suitable bases [strengths were calibrated to complement the thiophilicity of R^1^–SH]. Even though both the first and second steps of the drawn mechanism are nominally thiol-disulfide exchange reactions [albeit driven by loss of gaseous COS], the ratio κ = *k*_2_*/k*_1_ can range from 0.1 to 5 for neutral monofunctional aliphatic thiols (carbamoyl disulfide intermediates were observed in all cases), while with bifunctional thiols, where the second step can proceed intramolecularly because a cyclic disulfide is formed, κ > 100 (i.e., intermediate not observed). Note that while this Scheme is focused on deprotection chemistry, the same chemical transformations can be channeled for directed formation of disulfide bridges [115].

A comprehensive menu of thiols was evaluated for their efficacy in Dts removal, and a detailed mechanistic understanding was achieved (Scheme 7). Other reducing agents, including borohydrides (e.g., NaBH_4_ and NaCNBH_3_) and phosphines (e.g., Ph_3_P, nBu_3_P) in aqueous media^20^, were tested and shown to remove Dts as well^21^.

These studies set the stage for applications of Dts-amino acids for SPPS, as pursued primarily by Fernando Albericio during his productive 2-year postdoctoral stint (1983–1984) in Minneapolis, and continued by him on frequent visits whenever there was a break in his teaching schedule at the University of Barcelona. Not counting several preliminary communications at peptide symposia, our main results were reported in the *J. Am. Chem. Soc*. in 1985 [13] and in the *Int. J. Pept. Prot. Res*. in 1987 [44].

As alluded to earlier in this perspective (Scheme 2 and accompanying discussion), we described the first *three-dimensional* orthogonal protection scheme suitable for the preparation of fully and partially protected peptide segments. The scheme relied on thiolyzable Dts (acid-stable) for *N*^α^-amino protection, acidolyzable *tert*-butyl (stable to bases, thiols, and nucleophiles) for side-chain protection, and photolyzable *ortho*-nitrobenzyl (ONb) ester anchoring (acid-stable) as its three axes of orthogonality, and the leucine enkephalin sequence [H-Tyr-Gly-Gly-Phe-Leu-OH] was selected as the target for proof-of-concept experiments [13].

**Scheme 8:**
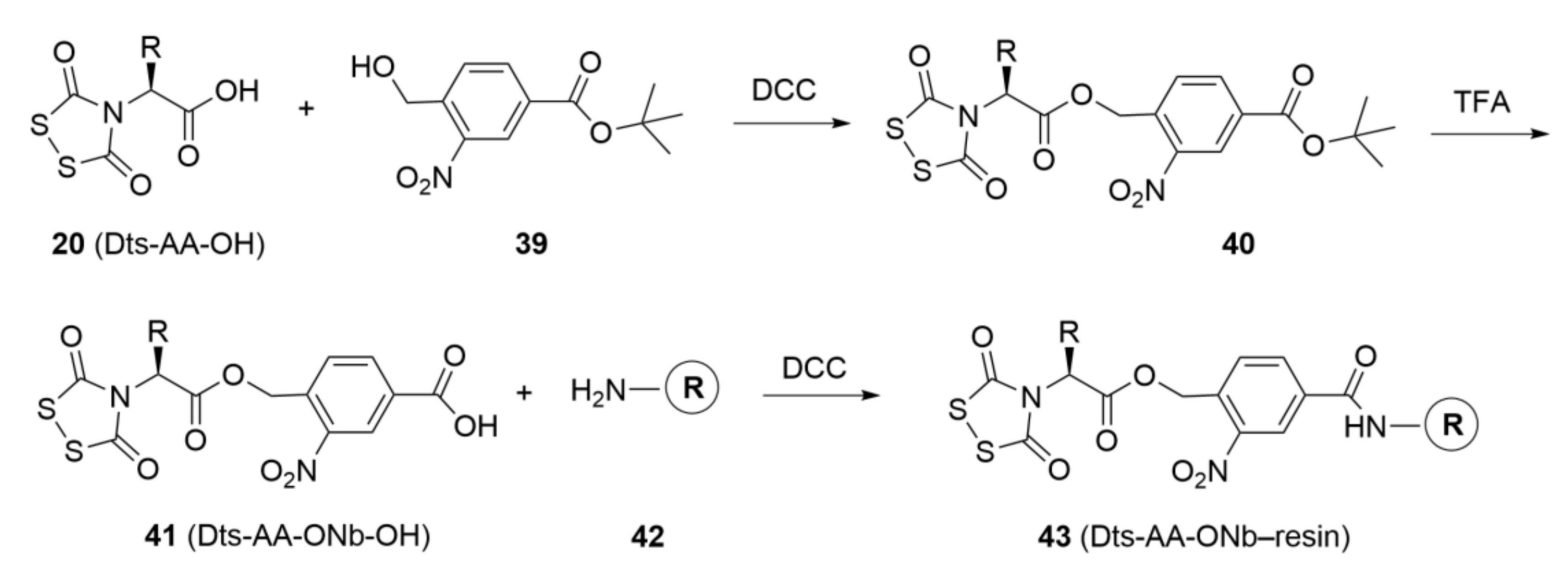
Preformed handle strategy for anchoring *N*^α^-Dts protected *C*-terminal amino acid residues as *ortho*-nitrobenzyl (ONb) esters [13].

Following a “preformed handle” strategy (Scheme 8), Dts-amino acids (**20**) were coupled to *tert*-butyl 4-(hydroxymethyl)-3-nitrobenzoate (**39**), as mediated by *N*,*N*′-dicyclohexylcarbodiimide (DCC), to provide intermediates **40**, which were treated with TFA to give crystalline 4-(*N*^α^-dithiasuccinoylaminoacyloxymethyl)-3-nitrobenzoic acids (**41**). Next, DCC-mediated couplings of **41** onto amino-containing supports (**42**) provided starting Dts-amino acid-handle–resins **43**, and chain elongations proceeded by cycles involving (i) deprotection with β-mercaptoethanol (0.5 m)–DIEA (0.5 m) in CH_2_Cl_2_ (2 × 2 min) and (ii) coupling with DCC in CH_2_Cl_2_ (90 min), confirmed to have reached completion by a negative ninhydrin test [116]. The resultant Dts-Tyr(tBu)-Gly-Gly-Phe-Leu-ONb–resin was then cleaved in several ways, resulting in four partially or fully deblocked leucine-enkephalin derivatives: Dts-Tyr(tBu)-Gly-Gly-Phe-Leu-OH, Dts-Tyr-Gly-Gly-Phe-Leu-OH, H-Tyr(tBu)-Gly-Gly-Phe-Leu-OH, and H-Tyr-Gly-Gly-Phe-Leu-OH. All of these were obtained in good yields and purities, and clearly establish all three dimensions of orthogonality. Importantly, the overall reaction sequences used were entirely *free* of racemization.

The mildness of the Dts removal conditions was emphasized in model experiments with a dipeptide–resin sequence known to be especially prone to intramolecular cyclization (diketopiperazine formation) under either acidic or basic conditions. Thus, Prot-d-Val-l-Pro-ONb–resin was assembled for Prot = Boc, Fmoc, and Dts. When Dts was removed by the preferred β-mercaptoethanol-containing cocktail already referred to, loss of chains from the support was negligible, whereas DKP formation was an issue (manageable, to some extent) with Boc chemistry (use of TFA for deprotection). Worst of the three, DKP formation when the protecting group was Fmoc (use of piperidine for deprotection) was so severe as to render ONb anchoring effectively incompatible with Fmoc chemistry.

**Scheme 9:**
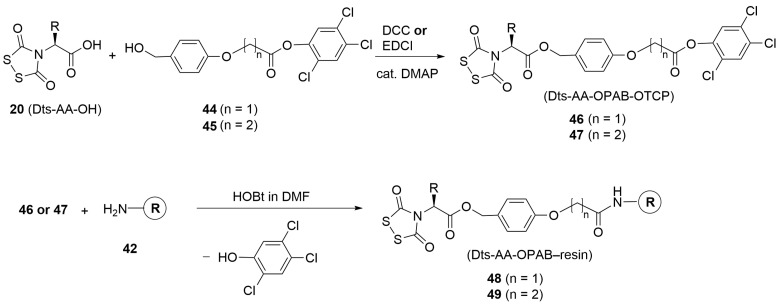
Preformed handle strategy [44] for anchoring *N*^α^-Dts protected *C*-terminal amino acid residues as *p*-alkoxybenzyl (PAB) esters.

Following up, we sought to further define the scope and limitations of Dts chemistry for mild orthogonal SPPS [44]. These studies relied on an acidolyzable *p*-alkoxybenzyl (PAB) ester anchoring linkage, again established by a “preformed handle” strategy (Scheme 9). Thus, Dts-amino acids (**20**) were coupled in solution onto 2,4,5-trichlorophenyl 4′-hydroxymethylphenoxyacetate (**44**) or the corresponding propionate (**45**), as mediated by DCC or 1-ethyl-3-(3-dimethylaminopropyl)carbodiimide (EDCI) in the presence of catalytic amounts of 4-dimethylaminopyridine (DMAP). The resultant derivatives (**46**, **47**) were purified and then coupled onto aminomethyl supports (**42**), using DMF as the solvent and HOBt (0.1 m) as a catalyst.

Stepwise chain elongation followed protocols introduced earlier, but were now extended with additional options for Dts removal, and expanding the repertoire of amino acid residues that could be added. While the previously described β-mercaptoethanol (0.5 m)– DIEA (0.5 m) in CH_2_Cl_2_ (2 × 2 min) recipe continued to be effective, further options were provided by a mixture^22^ of *N*-methylmercaptoacetamide (MAc) (0.5 m)–*N*-methylmorpholine (NMM) (0.5 m) in CH_2_Cl_2_ (2 × 2 min) **or** *N*-methylmercaptoacetamide (MAc) (0.5 m)–1-hydroxybenzotriazole (HOBt) (0.1 m) in *N*,*N*-dimethylformamide (DMF) (2 × 2 min). DCC-mediated couplings were carried out in CH_2_Cl_2_ wherever possible (up to 25% CH_3_CN was added as needed for solubility purposes), although some residues were added with DMF (containing 0.1 m HOBt) as the solvent. Completion of coupling reactions (>99.5%) was verified by negative ninhydrin (Kaiser) tests [116].

At the conclusion of chain assemblies, peptides were efficiently released from the PAB-linked supports by treatment with TFA, diluted as needed with CH_2_Cl_2_ or trifluoroethanol (TFE), and containing one or more appropriate carbocation scavengers like dimethyl sulfide and/or ethane-1,2-dithiol (EDT). 

**Scheme 10:**
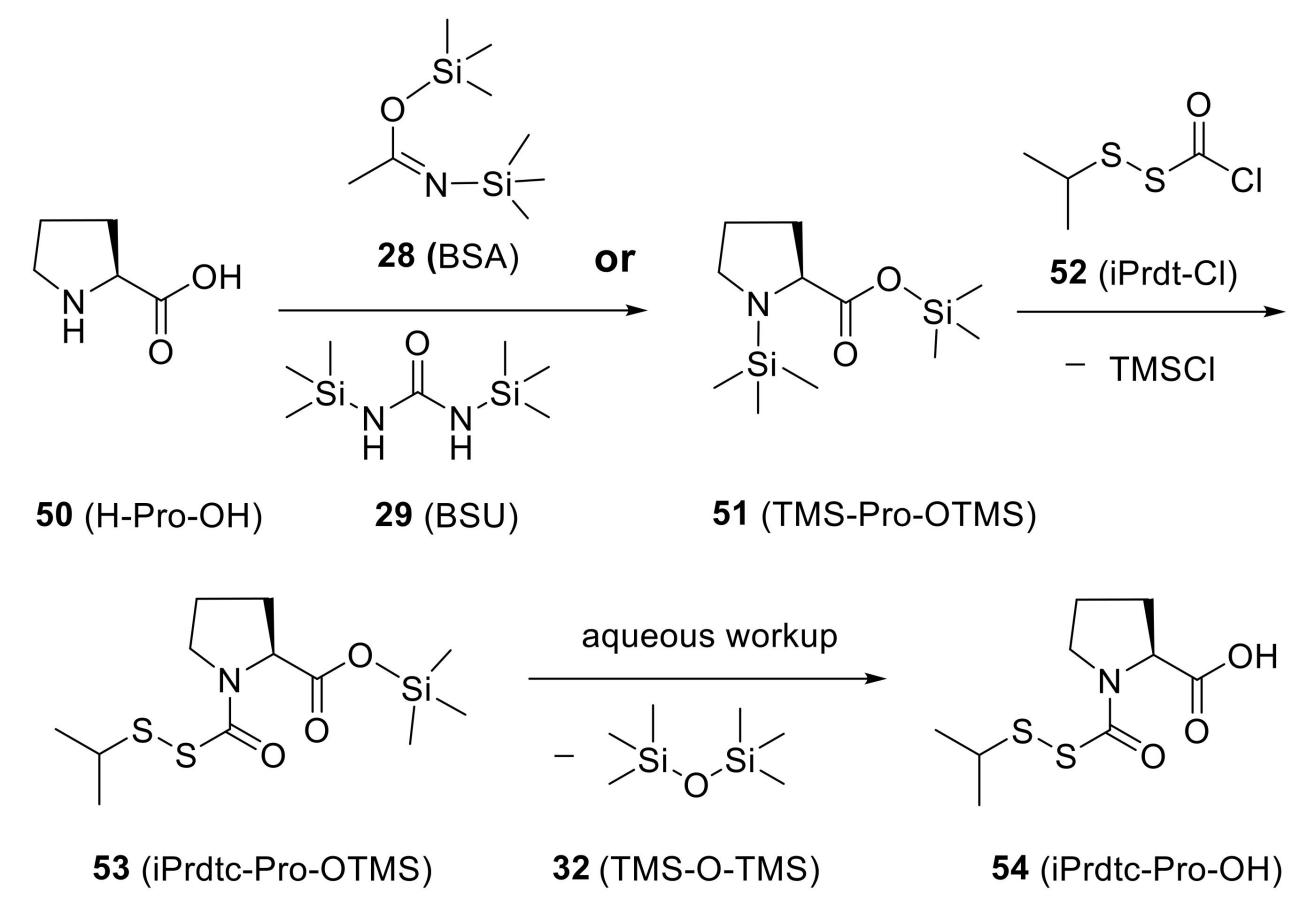
Preparation of *N*-(*iso*-propyldithio)carbonyl-l-proline (**54**), the open-chain carbamoyl disulfide-protected derivate of proline that complements Dts protection for the other 19 proteinogenic α-amino acids. The chemistry of carbamoyl disulfides was initially established when they were detected as intermediates in the thiolytic removal of Dts (Scheme 7 and accompanying references). The shown chemistry was developed by one of us (GB) towards the beginning of his independent professional career at the University of Minnesota [107]. Either BSA (**28**) or BSU (**29**) (this latter is a result from ref. [101]) can be used to create the bis(trimethylsilylated) derivative **51** *in situ*, whereupon it was acylated further with **52**. Alternatively (as described in ref. [107] but not drawn out in the present Scheme), we treated proline (**50**) with trimethylsilyl chloride (**55**, TMSCl) in CHCl_3_–CH_3_CN (4:1; 2 h reflux) to generate HCl^.^Pro-OTMS (**56**) *in situ*, which was then acylated with **52** in the presence of Et_3_N (2 equiv) at ~0 °C. Independent of our work [107], Wünsch and coworkers investigated (alkyldithio)carbonyl protection of α-amines [118].

A number of model peptides were prepared successfully by Dts SPPS, applying the aforementioned protocols. These peptides include Merrifield’s historically important tetrapeptide l-Leu-l-Ala-Gly-l-Val [35,117], methionine enkephalin (H-Tyr-Gly-Gly-Phe-Met-OH), and bradykinin (H-Arg-Pro-Pro-Gly-Phe-Ser-Pro-Phe-Arg-OH). Incorporation of proline (**50**) relied on the carbamoyl disulfide derivative **54**, which was prepared by using (*iso*-propyldithio)carbonyl chloride (**52**) to acylate proline (with temporary TMS protection established *in situ*) (Scheme 10). Side-chain protection was mostly provided by the standard TFA-labile tBu-type groups, but arginine was protected with a nitro (NO_2_) group which was removed in a separate catalytic hydrogenolysis step. 

Yields from SPPS were good to excellent, and purities were exquisite, including absolutely no evidence for racemization (<0.05%). Dts protection actually has a “*racemization-shielding*” effect, since the cyclic structure precludes formation of oxazolone-like species that are intermediates in the low-level racemization noted for urethane-type groups such as Boc or Fmoc.

**Scheme 11:**
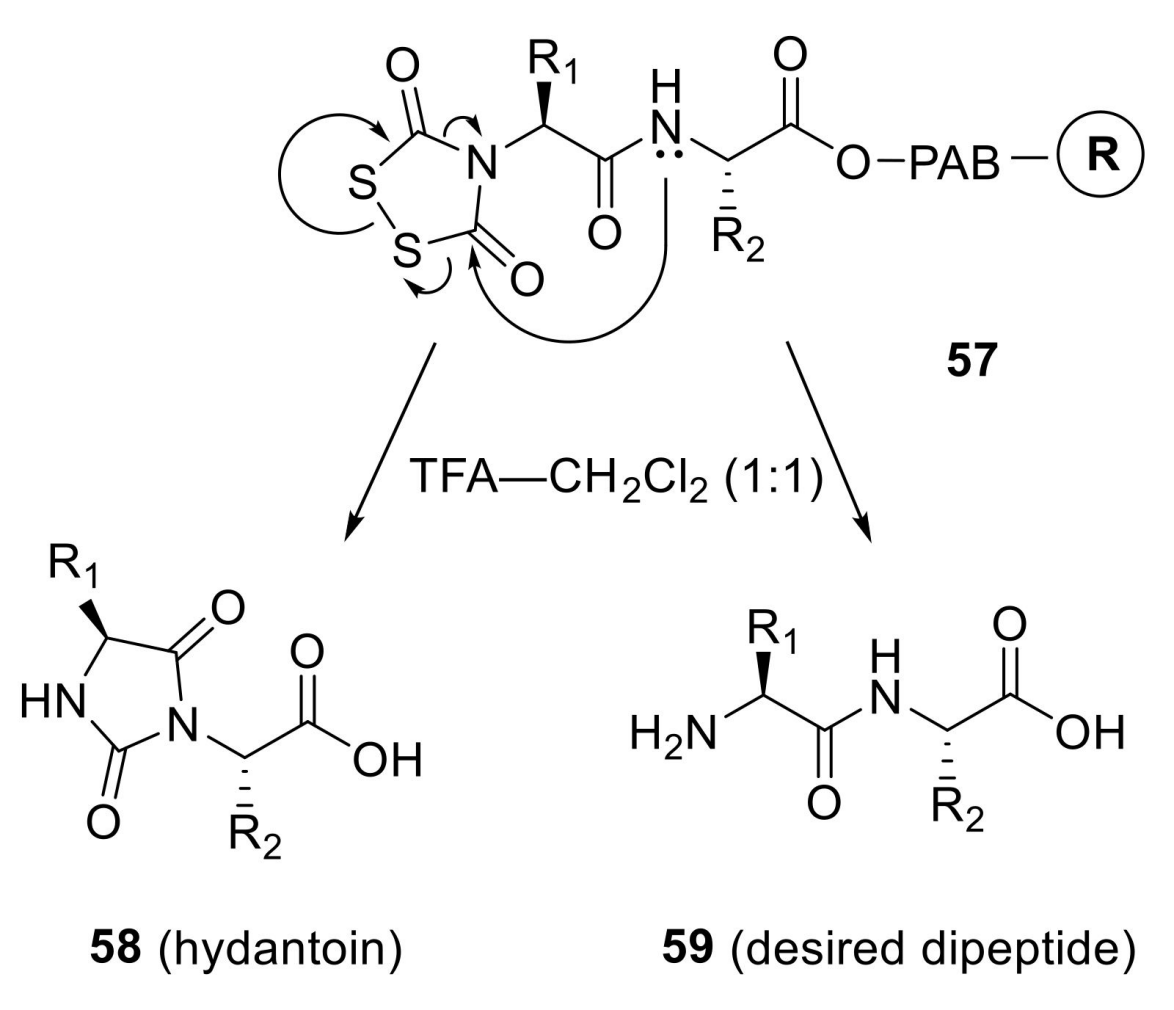
Susceptibility of the Dts-dipeptidyl unit to hydantoin formation. As described by Albericio and Barany [44], a resin-bound Dts-dipeptide **57** (see Scheme 9 for the anchoring strategy) was treated with Et_3_N (0.5 m) in CH_2_Cl_2_ for 15 min, following which the peptide products were released from the PAB anchor by TFA treatment to provide a mixture of hydantoin **58** and desired product **59** in a 3:2 ratio. In a separate experiment, **57** was treated with nBu_3_P (0.1 m) in CH_2_Cl_2_–CH_3_CN (3:1) to *quantitatively* give, after TFA cleavage, the *pure* hydantoin **58**.

Thus, our 1987 *Int. J. Pept. Prot. Res.* publication [44] supported an optimistic outlook for stepwise SPPS with Dts-amino acid chemistry, but did include one worrisome hint of a possible “Achilles heel” (Scheme 11). Thus, under *exaggerated conditions*, we showed that a model resin-bound dipeptide (**57**) underwent partial conversion to the corresponding hydantoin (**58**). Results were confirmed after TFA cleavage of the PAB anchor released into solution a mixture of the desired peptide (**59**) and the modified peptide (**58**), where the latter component matched an authentic standard made by a literature procedure.

Over the next several years, as we tackled more “difficult” sequences^23^^,^^24^, we noted that amino acids were no longer being incorporated in the desired stoichiometric ratios, as judged by quantitative amino acid analysis. Rather, there was a fall-off towards the *N*-terminus of these *C* → *N* syntheses. In a few cases (unpublished work), we isolated the culprit “terminated” peptides, and showed that they had masses consistent with hydantoin formation (*m*/*z* 26 amu higher than desired). With guarded optimism, we then launched efforts to evaluate novel resin supports and modified coupling protocols in the hope that they might yet lead to reliable stepwise SPPS by Dts chemistry, regardless of the target sequence.

## 6. Invention of PEG–PS Graft Resin Supports for SPPS and Other Applications

**Scheme 12:**
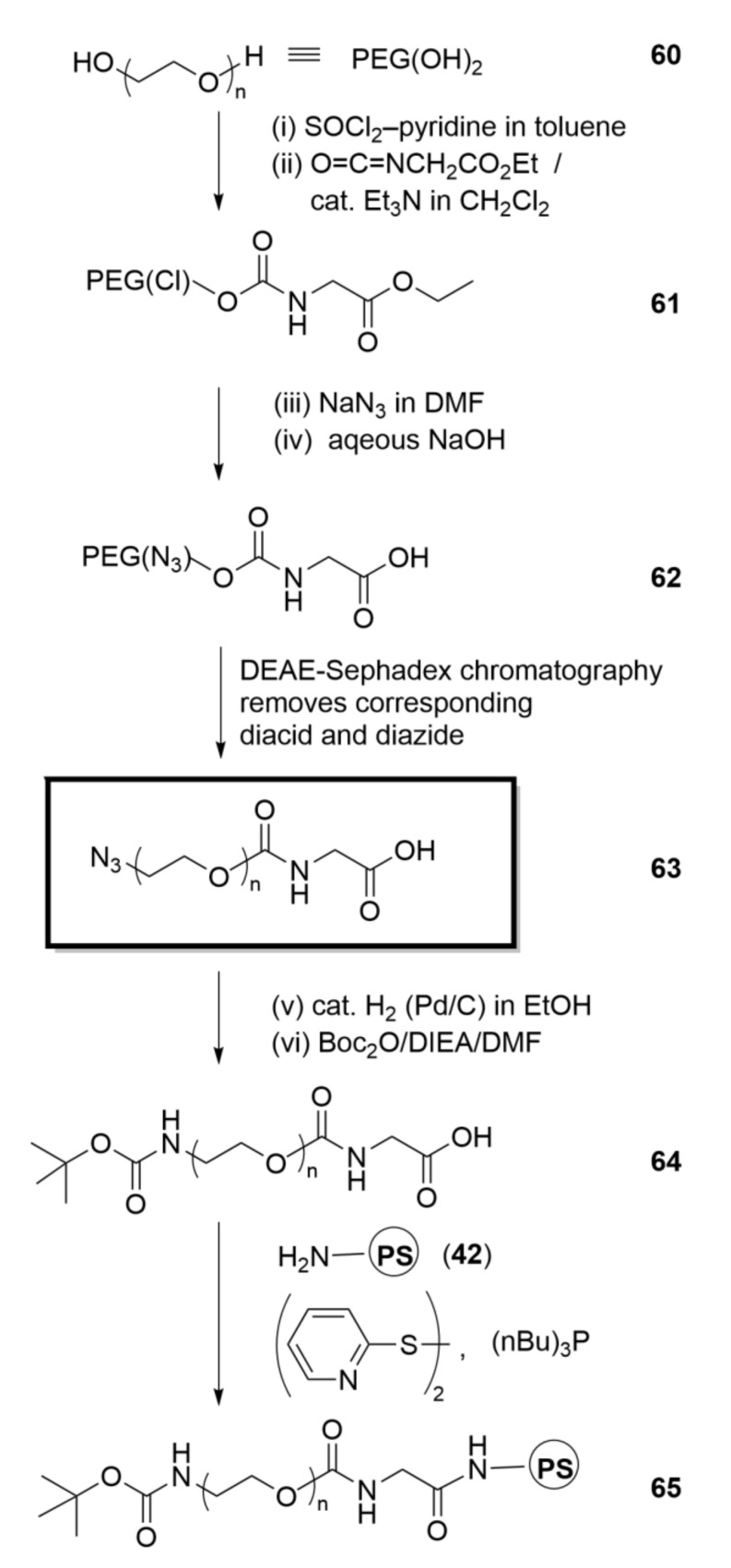
Preparation of “first generation” PEG–PS supports (**65**). Using bifunctional PEG (**60**) as a starting material, the protected heterobifunctional PEG “large” amino acid **64** was created in six chemical and one ion-exchange purification steps by the sequence shown. Step (i) is *intentionally* carried out with substoichiometric levels of reagents, so as to create a product that is a statistical mixture of starting diol, a monochloride, and a dichloride. All subsequent chemical transformations are quantitative. Compounds **61** and **62** are also statistical mixtures, whereas the boxed compound **63** has a defined structure exactly as drawn. Once **64** is grafted onto amino-functionalized supports [Mukaiyama oxidation-reduction shown here, but other methods can be used as well], it can be applied further for stepwise SPPS according to a range of chemistries (primarily Fmoc, but also Boc and Dts). For example, the Boc group can be removed with TFA–CH_2_Cl_2_ (1:1), followed by neutralization with DIEA–CH_2_Cl_2_ (1:19), then introduction of an “internal reference” amino acid (IRAA), and finally introduction of a carboxyl group-containing handle like Fmoc-AA-OPAB or Fmoc-PAL that serves as the starting point for SPPS. The indicated chemistry was carried out both for *n* ≈ 45 (PEG-2000) and *n* ≈ 90 (PEG-4000). Adapted from ref. [125].

An earlier section of this perspective describes a key control experiment we carried out at a time when we sought a fundamental understanding of the chemistry underpinning our PEG-based “functional purification” routes to Dts-amino acids at the level of purity required for them to be useful for SPPS. Thus, we showed that ion-exchange chromatography of *mixtures* of PEG derivatives could be used to isolate a *specific* pure heterobifunctional PEG. Samuel Zalipsky then had the important insight that we harness these discoveries to prepare the pure PEG derivative **64**, which is effectively a “big” protected amino acid (Scheme 12). Subsequently, Zalipsky collaborated with Fernando Albericio to couple **64** onto aminomethyl-polystyrene, thereby creating the first PEG–PS graft support (**65**). With the average molecular weight of PEG and the initial loading of amino groups on PS that were used, this PEG–PS turned out to be *equal* amounts PEG and PS, on a *w*/*w* basis.

Albericio then used Dts chemistry with PAB anchoring to assemble, on “first generation” PEG–PS, the Merrifield model tetrapeptide, which was obtained in an extraordinary purity of 99.6%, in the *same hands* that had earlier obtained a purity of 98.8% on PS. In Zalipsky’s independent hands, SPPS on PEG–PS also gave a higher purity than on PS, the only problem being that “Albericio on PS” was slightly better than “Zalipsky on PEG–PS”. We did not quite know what to do with this, but dutifully communicated our findings at the 9^th^ American Peptide Symposium, held in 1985 in Toronto, Canada [126]. Our working hypothesis was that somehow a *spacer* effect of PEG was responsible for the incrementally better SPPS results with PEG–PS.

Zalipsky graduated and went on to have an impactful career in the biotechnology sector while, back in Minneapolis, GB was left to ponder how to bring this work to the next level. Clearly, ion-exchange chromatography to purify heterobifunctional PEG derivatives that were, in turn, created by multi-step chemical routes starting with homobifunctional PEG (two OH end groups), was not likely to be amenable to scale-up. 

Through the contributions of two hard-working postdoctoral fellows—Jane Chang, a University of Illinois-trained organic chemist who joined GB´s laboratory in late 1988; and Núria Solé, who arrived in 1990 from Barcelona, Spain—and with the further collaboration of Derek Hudson and his team at Biosearch in San Rafael, California, we pursued several simplifications and improvements: (i) using a commercial product called Jeffamine (like PEG, except two H_2_N- end groups) as a starting point; (ii) reacting Jeffamine with maleic anhydride to create a polymeric intermediate that could be coupled^25^ onto aminomethyl–PS or 4-methylbenzhydrylamine (MBHA)–PS, and then applying mild acid-catalyzed hydrolysis conditions, based on the protein chemical modification literature [127], to release free amino groups on the resultant PEG–PS; (iii) reacting Jeffamine with succinic or glutaric anhydride (in addition to maleic anhydride), then proceeding as before, but ultimately using coupling reactions with 1,2-ethylenediamine to establish the needed free amino groups on PEG–PS; (iv) in a totally different approach, coupling orthogonally protected *N*^α^-Fmoc, *N*^δ^-Boc-ornithine onto aminomethyl–PS, then removing the Fmoc group selectively, and then coupling a carboxymethylated derivative of commercially available monofunctional poly(ethylene glycol) methyl ether (MPEG), i.e., CH_3_O(CH_2_CH_2_O)_45_CH_2_CO_2_H, onto the δ-amino side-chain of ornithine (Orn, which also doubles as an IRAA); after all this, using the α-amino group of Orn as the starting point for addition of appropriate handles and for stepwise Fmoc SPPS.

The work summarized in the preceding paragraph was patented (see below) and eventually published and/or reviewed [125,128,129], albeit sometimes with a considerable time lag from when it was first explored in our Minneapolis laboratories. Please note that for some of these formulations, but most dramatically for (iv), we pivot from the *spacer* model to an *environmental* model as an explanation for the beneficial effects conferred by the PEG component. Furthermore, since our methods incorporate PEG chains in a *predefined size range*, they offer considerable flexibility for adjusting the PEG:PS ratio, as well as the initial loading of functional groups that are the starting point for subsequent SPPS. 

Regardless of how it is formulated, PEG–PS proved to have several useful properties that differentiate it from PS. Thus, PEG–PS is compatible with an extended array of solvents, e.g., acetonitrile, that go beyond the chlorinated hydrocarbons (e.g., CH_2_Cl_2_) and polar amides [e.g., *N*,*N*-dimethylformamide (DMF), *N*,*N*-dimethylacetamide (DMA), and *N*-methyl-2-pyrrolidinone (NMP)] used with PS; PEG–PS is resistant to high pressures, allowing its use in flow-through reactors that represent an attractive alternative to the batch-based methods that are the norm when working with PS; and PEG–PS facilitates assorted biological assays, as well as treatments with enzymes, that must be carried out in aqueous milieus. 

In critical experiments, we investigated peptides such as acyl carrier protein (ACP) fragment 65–74, which is widely understood to be “difficult” (see footnote 23, earlier in this perspective), and found that Fmoc SPPS on PEG–PS gave *significantly* better results, by comparison to PS. Furthermore, we were able to carry out, with PEG–PS supports, successful SPPS experiments in which acetonitrile was used as the solvent for all couplings and washings; the control experiments on PS failed to incorporate amino acids, let alone yield the desired final peptide products.

PEG–PS was patented in 1993 and licensed to Biosearch, under whose auspices (and the auspices of successor companies such as MilliGen/Biosearch, Millipore, PerSeptive Biosystems, Applied Biosystems, and ThermoFisher) it became a blockbuster product for peptide synthesis, netting millions of dollars in sales (and a not-insubstantial royalty back to the University of Minnesota). As the state-of-the-art standard support for stepwise Fmoc SPPS^26^, particularly for continuous-flow automated synthesizers, PEG–PS was central to numerous important scientific undertakings, including our own studies on bovine pancreatic trypsin inhibitor (BPTI) and its analogues designed to study protein folding [74,130].

While we were playing with PEG–PS, the research group of Ernest Bayer in Tübingen, Germany, used hydroxymethyl-polystyrene as an initiator to polymerize ethylene oxide, thereby creating their own variant of PEG–PS [131,132]. One of Bayer’s students, Wolfgang Rapp, founded a small company in 1988 that was able to reproducibly prepare their materials on large scales, whereupon they were branded as “TentaGel“ and have been a staple support for much SPPS, and allied efforts, ever since. 

We found no substantive performance differences between commercial TentaGel and commercial PEG–PS. In fact, our “shaving” studies, performed by Josef Vágner in Minneapolis under a contract with Michal Lebl and his team at the Selectide Corporation in Tucson, Arizona, used TentaGel beads. In this project, which was announced in a 1996 *Proc. Natl. Acad. Sci. USA* paper [133], we took advantage of the fact that proteolytic enzymes such as chymotrypsin, elastase, or pepsin could only cleave peptide sequences near the *surface* of these beads, and then applied a novel orthogonal scheme to generate both biological test sequences on the *surface* and “coding” sequences in the *interior*.

## 7. A Watershed Visit

In October 1991, GB had been recently promoted to the rank of full Professor at the University of Minnesota and was thriving with a modest-sized, well-funded, talented research group at work, and two small children at home. Quite unexpectedly, he was invited by one *Morten Meldal*—not a name he recognized at the time—for the honor of delivering a “Frontiers in Science” lecture at the Carlsberg Research Laboratory in Copenhagen, Denmark. Accepting with delight, GB headed to the Minneapolis airport on Wednesday afternoon, 9 October, right after finishing that week’s teaching (an examination was scheduled to be administered *in absentia* on Friday), for a flight to Newark airport. After several hours on the ground (the Minnesota Twins playoff game^27^ against the Toronto Blue Jays was showing on the airport TV monitors), GB took an overnight overseas flight.

Meldal picked up GB at the Copenhagen airport and brought him directly to the lecture hall, whereupon he regaled an audience of about a hundred students and scientists for the next several hours^28^, with just a single intermission. Many of the topics making up the first portion of this perspective were covered, including a detailed progress report on Dts chemistry and our up-to-date understanding of why PEG–PS, in its various formulations, was such a useful support—a relatively fresh point of view at the time.

Some details of the Copenhagen visit remain etched in GB’s memory to this very day: Morten’s keen intellect and curiosity along with his kind hospitality and personal generosity; insightful conversations with Klaus Bock and others at Carlsberg as a follow-up to the lecture and continuing for a second day; the gift of a coffee table size book about the historic scientific legacy of the institution [134,135], which includes introduction of the pH scale by Søren P.L. Sørensen (1868–1939) and fundamental protein biophysical chemistry from Kaj Linderstrøm-Lang (1896–1959) and his school; a photo-op in front of the building where Niels Bohr was born^29^ in 1885; an initial meeting and delightful dinner with Meldal’s first graduate student at the Carlsberg Laboratory, Knud Jensen, in whom GB planted the seed to eventually pursue postdoctoral studies in Minneapolis; accommodations at the elegant Hotel 3 Falke (now Falkoner) which has a concert hall as part of the complex (GB was able to score a ticket to one of his favorites, Verdi’s rarely performed opera *Nabucco* with its famous chorus of the Hebrew slaves); returning to the hotel room just before midnight local time on Friday night and watching CNN (the only English language channel available) until almost dawn Saturday^30^; being driven through town for some sightseeing which included the Little Mermaid statue in the harbor and the Tivoli fairgrounds. 

More than thirty years later, PRH still remembers being in the audience that initial Thursday mid-morning, and how GB’s visit inspired him to travel to Minneapolis for his own graduate studies.

## 8. Invention of PEGA Resin for SPPS and Other Applications^31^

**Figure 1:**
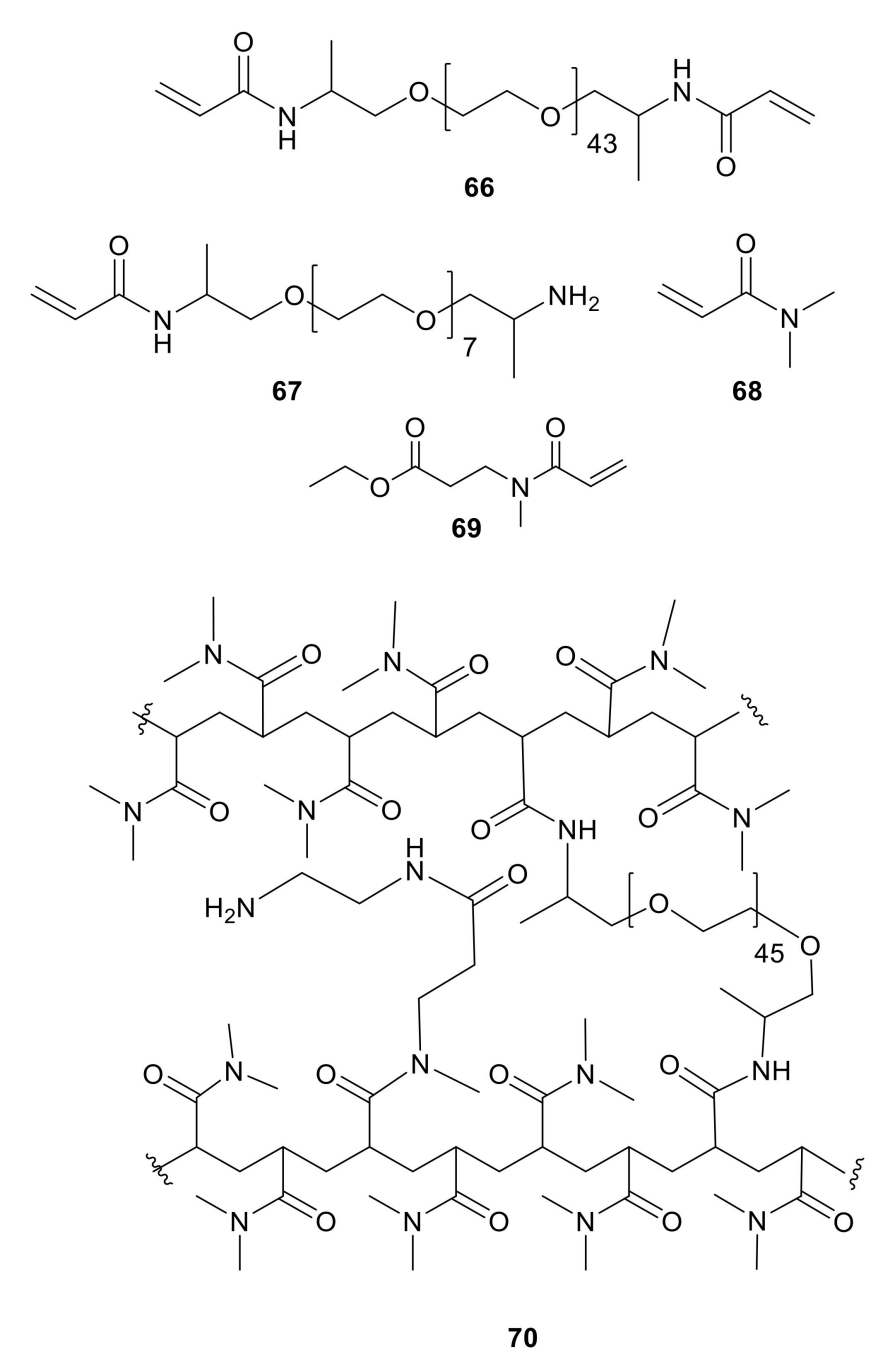
Chemistry underlying PEGA [polyethylene glycol-poly-(*N*,*N*-dimethylacrylamide) copolymer] supports. In Meldal’s original report [136], monomers **66**, **67**, and **68** were copolymerized. Note that **67** provides (right side of structure, as drawn) an amino functionality for subsequent synthesis. A few years later, Renil and Meldal [137] developed a *modified* PEGA, again using **66**, but now adding acryloyl sarcosine ethyl ester (**69**) in place of **67**. In this case, the needed amine functionality was established by treating the polymer with 1,2-ethylenediamine, resulting in a support (**70**) that was suitable for SPPS and SPOS.

Within half a year of GB’s visit to Copenhagen, Meldal submitted to *Tetrahedron Letters* a report on the design and preparation of what he termed a “PEGA Resin” [136]. PEGA was prepared in both a granulated and a beaded form by copolymerization of bis-2-acrylamidoprop-1-yl-PEG-1900 (**66**), 2-acrylamidoprop-1-yl[2-aminoprop-1-yl]-PEG-300 (**67**), and *N*,*N*-dimethylacrylamide (**68**) (Figure 1). The resultant PEGA (Figure 1, including legend) showed good swelling in DMF, CH_2_Cl_2_, TFA, alcohols, and H_2_O, and offered several desirable features such as flow stability, high polarity to assist peptide solvation, and transparency (due to the absence of aromatic groups) that would allow direct spectroscopic monitoring of materials bound to the resin.

To establish the value of PEGA (as initially formulated) for Fmoc SPPS, Meldal carried out a synthesis of ACP 65–74, a well-accepted challenging target [see discussion of PEG–PS earlier in this perspective]. The Copenhagen experiments used the Rink linker [138] for anchoring, and the building blocks were Fmoc-amino acid 3,4-dihydro-3-hydroxy-4-oxo-1,2,3-benzotriazine (Dhbt-OH) esters, which allowed for coupling reactions to be monitored spectrophotometrically [139]. Reaction times were 2 to 70 min, significantly faster than in a control synthesis on a kieselguhr-supported polyamide resin (Pepsyn K) with which Meldal had become familiar during his postdoctoral work with Bob Sheppard at Cambridge. Most notably, the notoriously difficult coupling of Val^65^ to Gln^66^ required 65 min to reach completion with PEGA, whereas with Pepsyn K, it was still incomplete after 1140 min.

**Scheme 13:**
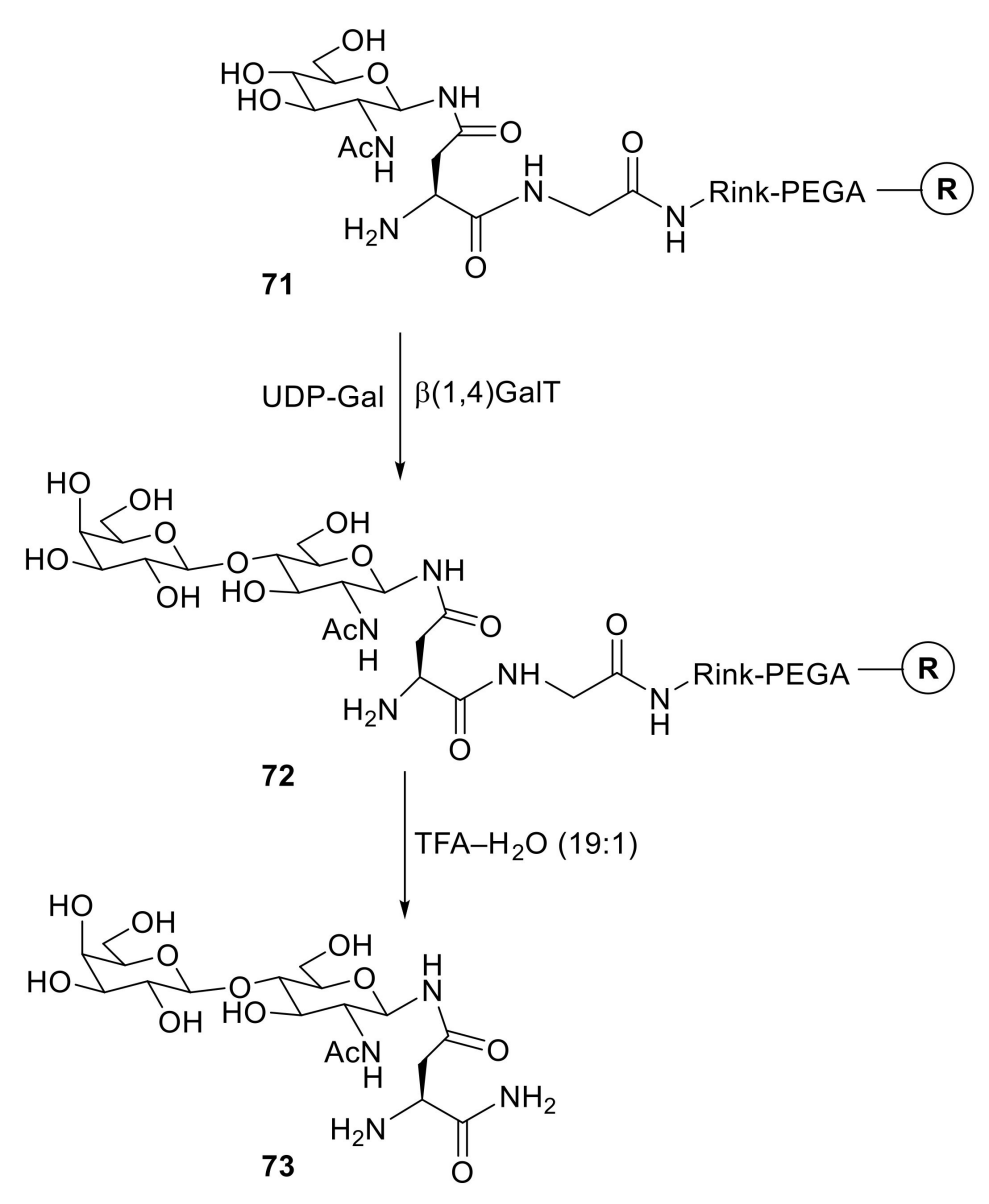
Solid-phase glycopeptide synthesis on PEGA, featuring on-resin enzymatic steps [140]. This publication also explored a variation where **71** was protected with *N*^α^-Fmoc. In this latter case, on-resin sugar transfer mediated by the enzyme did occur, but not nearly as efficiently.

Meldal and coworkers subsequently demonstrated that PEGA resins are permeable to enzymes [140]. In an important study (Scheme 13), Asn(*β*-d-GlcNAc)-Gly-Rink–PEGA [**71**, as drawn with a free amine or the corresponding structure with *N*^α^-Fmoc protection] was glycosylated using uridine 5′-(*α*-d-galactopyranosyl dihydrogen diphosphate) (UDP-Gal) as the donor and bovine *β*-(1→4)-galactosyltransferase as the enzyme. The reaction showed >95% conversion into resin-bound product **72** within 48 h. Cleavage with TFA–H_2_O (19:1) gave Asn(β-*N*-acetyllactosamine)-Gly-NH_2_ (**73**) as the final product.

A follow-up paper in *Proc. Natl. Acad. Sci. USA* described a solid-phase assay for mapping the active site of subtilisin Carlsberg [141]. A large peptide library with the general format Tyr(NO_2_)-X^5^X^4^-Pro-X^3^X^2^X^1^[Lys(ABz)-spacer–PEGA] was prepared, installing 2-aminobenzamide (ABz) [coupled as Fmoc-Lys(Boc-ABz)-OPfp] and 3-nitrotyrosine [coupled as Fmoc-Tyr(NO_2_)-OH] as a donor-acceptor pair. The library was then incubated with subtilisin at either pH 8.5 or pH 5, based on the idea that resin-bound peptides containing both ABz and Tyr(NO_2_) would not be seen under a fluorescence microscope. On the other hand, when the resin-bound peptide sequence was a subtilisin substrate, the relevant beads would have only the ABz fluorophore, and be illuminated. The model substrate was H-Tyr(NO_2_)-Phe-Gln-Pro-Leu-Asp-Glu-Lys(ABz), which was 80% cleaved after 1 h and quantitatively cleaved after 24 h. Several subtilisin-sensitive sequences were found, with conversion ranging from 3 to 44% in 60 min.

## 9. Dts Chemistry in the Service of Glycopeptide Synthesis

In the mid-1990s, there was a convergence of concepts and experimental accomplishment achieved independently in Copenhagen and Minneapolis^32^. Morten Meldal, working with Carlsberg Laboratory postdoctoral fellow Ernst Meinjohanns (co-advised by Klaus Bock), was motivated to synthesize *N*-acetyl-β-d-glucosamine glycosides [142] while GB was looking for new applications of the Dts protecting group and surmised that Knud Jensen, an Alfred Benzon Foundation postdoctoral fellow who had been Meldal’s first graduate student at the Carlsberg Laboratory, had the skillset to move the project forward. He was later joined by one of us (graduate student PRH, from Copenhagen) and postdoctoral fellow Divakaramenon Venugopal from India.

**Figure 2:**
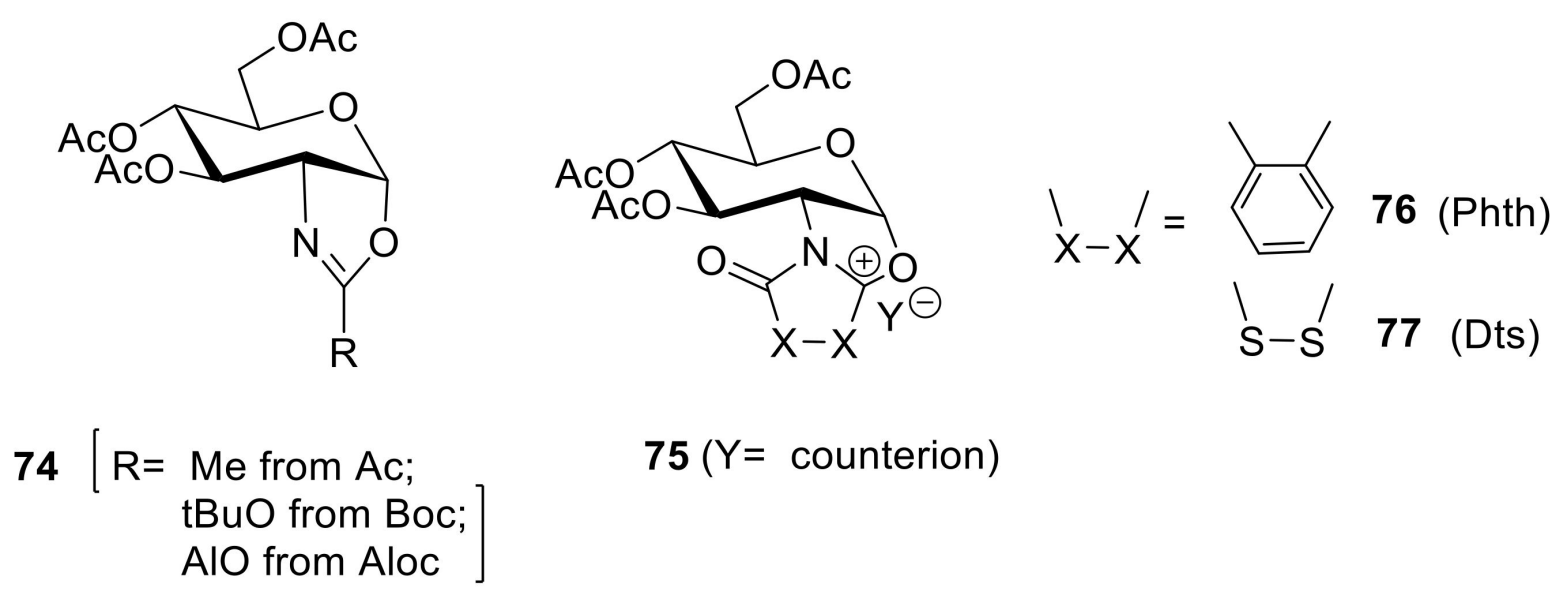
Oxazolines (**74**) or oxazolinium (**75**) intermediates formed from d-glucosamine derivatives during glycosylation. The precise chemistry followed depends on the nature of the functionality protecting the 2-amino group, as discussed later in the text.

**Scheme 14:**
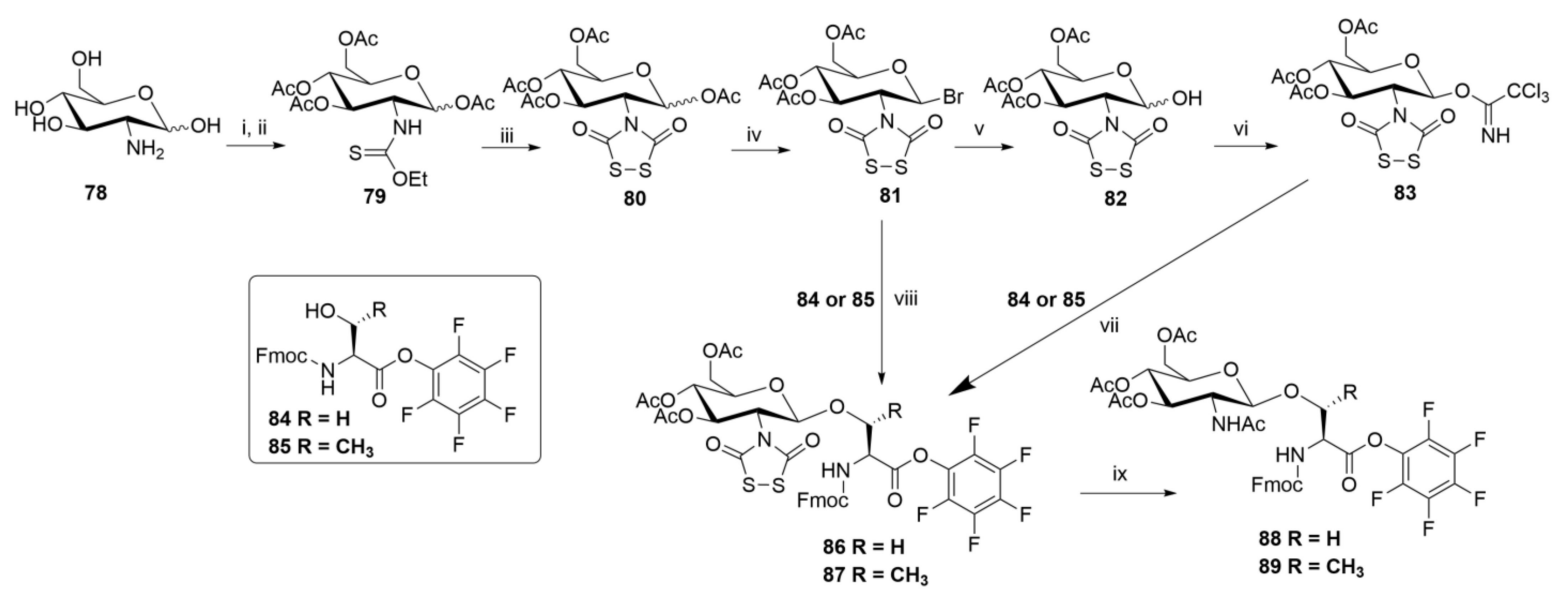
Synthesis of protected glycosylated derivatives of serine and threonine. Reagents/conditions: (i) EtO(C=S)SCH_2_CO_2_H in MeOH (Meldal) or [EtO(C=S)]_2_S in aqueous EtOH, pH 9–10 (Barany); (ii) Ac_2_O–pyridine (optionally diluted with CH_2_Cl_2_); (iii) Cl(C=O)SCl in CH_2_Cl_2_; (iv) 30% HBr in HOAc (optionally diluted with CH_2_Cl_2_); (v) H_2_O–acetone (1:3); (vi) CCl_3_CN–K_2_CO_3_ in CH_2_Cl_2_; (vii) AgOTf in CH_2_Cl_2_; (viii) AgOTf in CH_2_Cl_2_ in the presence of 3-Å sieves; (ix) Zn in HOAc–THF (1:9), followed 1 min later by 2 vol of neat Ac_2_O (the reduction/acylation conditions were discovered in Minneapolis [143] and adopted in Copenhagen [144]).

Both research groups successfully made the SPPS building blocks *N*^α^-Fmoc-Ser(Ac_3_-β-d-GlcNDts)-OPfp (**86**) and *N*^α^-Fmoc-Thr(Ac_3_-β-d-GlcNDts)-OPfp (**87**) by relatively similar multi-step routes starting from d-glucosamine hydrochloride (**78**) (Scheme 14 which consolidates findings from refs. [142] and [143]). The use of Dts in this manner was a significant innovation because, when working directly with *N*-acetyl-d-glucosamine (GlcNAc) derivatives or when the 2-amino group is protected by a urethane (e.g., Boc, Aloc, etc.), the appropriate glycosyl donors are readily converted to unreactive oxazoline intermediates (see structure **74** in Figure 2). On the other hand, when the 2-amino group is protected by two acyl-type groups (i.e., bis(protection) like *N*,*N*-diacetyl) or, even better, by a bivalent protecting group (e.g., Phth, **76** in Figure 2) that covers both free valences of the nitrogen, oxazolines cannot form. Instead, oxazolinium ions (see structure **75** in Figure 2) are formed, which are highly reactive and continue to give exclusively 1,2-*trans*-glycosides. Ostensibly, this would be a good outcome, were it not for the fact that harsh final deprotection conditions [e.g., hydrazine hydrate in EtOH–H_2_O (9:1) aqueous alcohol, 16 h reflux] are accompanied by undesired β-elimination of the glycan from Ser or Thr. As shown independently by both the Copenhagen and Minneapolis groups, all of these problems are neatly circumvented by using Dts protection (**77** in Figure 2).

The required building blocks (Scheme 14) were created by taking advantage of previously published chemistry that has been summarized earlier in this perspective. Thus, either of the two recommended reagents for introduction of the Etc group (see Scheme 4A, earlier) followed by peracetylation with acetic anhydride in the presence of pyridine gave intermediate **79** in excellent yield and purity. Next, (chlorocarbonyl)sulfenyl chloride (**5**) was applied to create [ZWK reaction; see Scheme 4A], in high yield with no observable by-products, the Dts heterocycle in compound **80**—this was obtained as an anomeric mixture favoring α over β by anywhere from 3:1 to 5:1. There followed treatment with hydrogen bromide (HBr) in acetic acid (HOAc) to establish cleanly the corresponding glycosyl bromide **81**, also an anomeric mixture but now favoring β over α. This result is unexpected, because α-bromides normally predominate due to the anomeric effect which stabilizes the axial (*α*) anomeric substituent; in this case, steric effects from Dts override the anomeric effect, resulting in preferential formation of the *β*-bromide.

In our hands, **81** reacted *directly* with Fmoc-Ser-OPfp **84** or Fmoc-Thr-OPfp **85**, in the presence of silver trifluoromethanesulfonate (AgOTf), to provide desired building blocks **86** or **87**, whereas Meldal concluded that **81** was too unstable to purify by silica gel chromatography and therefore used two more steps: hydrolysis to give hemiacetal **82**, followed by treatment with trichloroacetonitrile, to give β-trichloroacetimidate **83** (exclusively the β anomer, as proven by NMR), before again coupling in the presence of AgOTf to obtain the same building blocks **86** or **87**.

A further aspect of these programs was to develop and optimize conditions for removal of the Dts group that protected the 2-amino function of sugars [and subsequently to carry out *N*-acetylation of that liberated amino function]. In experiments using **80** (β anomer) as substrate, Meldal adapted conditions that we had reported previously (see earlier in this perspective) and focused on: (i) β-mercaptoethanol (0.2 m)–DIEA (0.5 m) in CH_2_Cl_2_ (87% yield); (ii) dithiothreitol (0.3 m)–DIEA (0.1 mm) in CH_2_Cl_2_ (98%); and (iii) NaBH_4_ (2 mol equiv) in CH_2_Cl_2_–MeOH (1:1) (94%).

Our independent work, using **86** or **87** as substrates, confirmed that standard thiolytic removal conditions, specifically β-mercaptoethanol–DIEA in CH_2_Cl_2_ or *N*-methylmercaptoacetamide (MAc) in DMF, efficiently removed Dts, but also displaced the pentafluorophenyl (Pfp) esters to form the corresponding thioesters. To circumvent these issues, we developed a novel procedure for simultaneous Dts removal and *in situ N*-acetylation, using zinc in HOAc–THF (1:9), followed 1 min later by 2 vol of neat Ac_2_O, and were able to produce *N*^α^-Fmoc-Ser(Ac_3_-β-d-Glc*p*NAc)-OPfp (**88**) and *N*^α^-Fmoc-Thr(Ac_3_-β-d-Glc*p*NAc)-OPfp (**89**) in yields of 80% and 87%, respectively [step (ix) in Scheme 14].

The Dts-protected glycosylated Pfp ester building blocks **86** and **87** were used by both research groups for the Fmoc SPPS of a variety of glycopeptides ranging from 6 to 12 amino acid residues in length [143,145,146]. Very similar strategies were adopted, with relatively minor differences in the details of the reaction conditions and choices of starting linker–resin support [i.e., Rink-PEGA or HMPA–Macrosorb SPR in Copenhagen versus PAL-Nle–PEG–PS in Minneapolis].

The Minneapolis team added the building block (3 equiv) as a DMF solution in the presence of HOBt (1.5 equiv), while the Copenhagen group did the same but in the presence of Dhbt-OH^33^ (1 equiv). Once safely incorporated within the growing peptide–resin, the Dts group was removed by thiolytic deprotection using previously reported cocktails, followed immediately by on-resin *N*-acetylation of the released 2-amino group of the sugar [Ac_2_O in DMF (Copenhagen) or CH_2_Cl_2_–pyridine (1:1) (Minneapolis)]; in this way, the Dts group was never exposed to piperidine used to remove the Fmoc group in subsequent cycles. Final release of glycopeptides was achieved with TFA–H_2_O (19:1), and final deacetylation occurred smoothly with methanol in the presence of a catalytic amount of sodium methoxide, which was neutralized with dry ice (CO_2_) after 15 min.

Additional studies reported from Copenhagen are interesting and significant. Mucins are glycoproteins that are found on the apical surface of epithelial cells, which are modified by clusters of *O*-glycosylation. These molecules have numerous functions including modulation of intracellular trafficking, regulation of half-lives of chemokines, and homing of leukocytes to inflammation sites; they are also associated with tumor progression and prognosis [147].

**Scheme 15:**
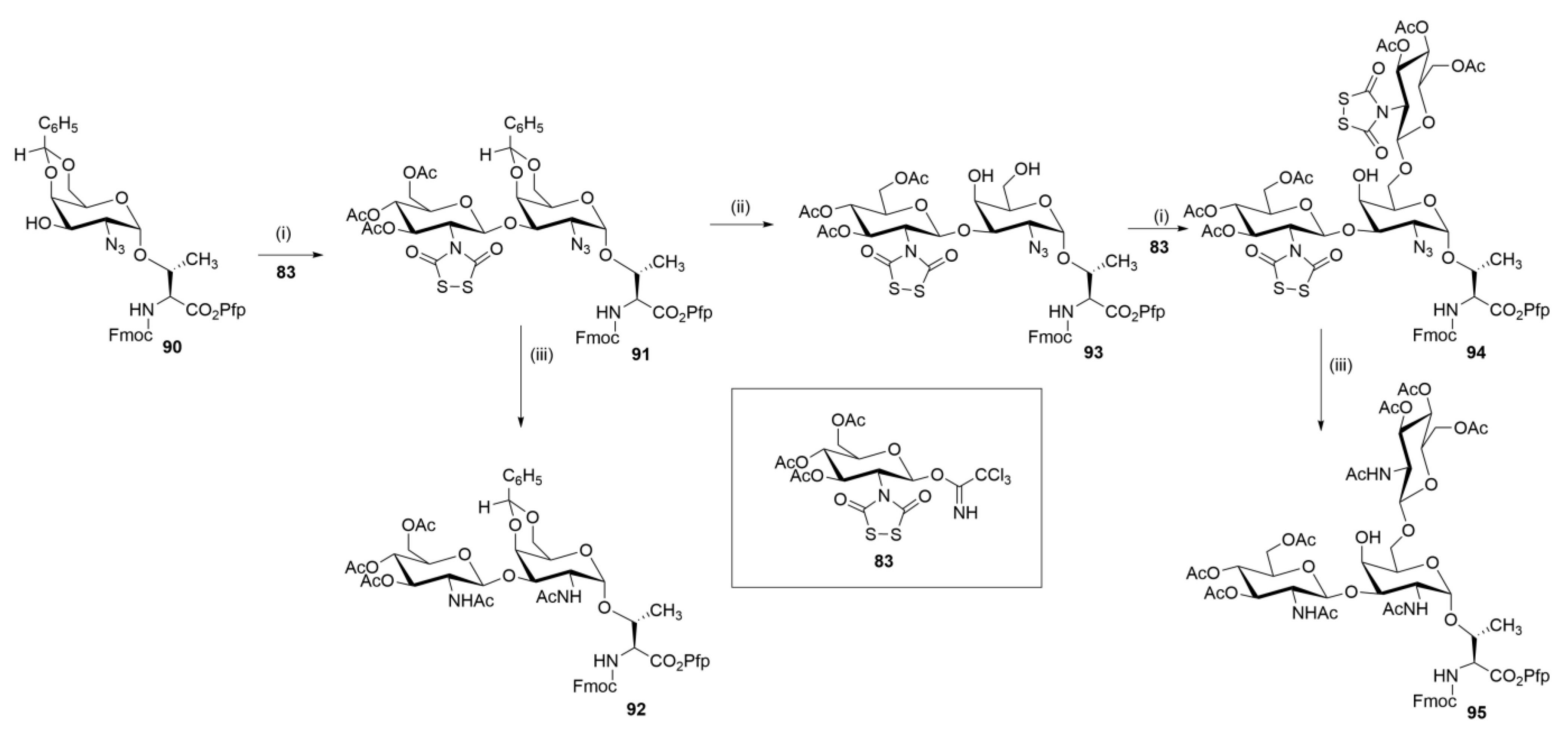
Synthesis of mucin core 3 building block **92** and mucin core 4 building block **95**. Conditions: (i) TMSOTf in CH_2_Cl_2_; (ii) HOAc–H_2_O (4:1) (70 °C; (iii) Zn in Ac_2_O–HOAc–THF (2:1:3).

Meldal and coworkers applied Dts chemistry to the synthesis of core 1, core 2, core 3, and core 4 mucin *O*-glycopeptides (Scheme 15). The core 3 building block **92** was synthesized in two steps by first coupling Dts-protected β-trichloroacetimidate **83** with the Fmoc and 4,6-*O*-benzylidene protected amino acid sugar **90**, using TMSOTf as a promotor, and then subjecting the resultant **91** to simultaneous reduction and *N*-acetylation with zinc in a mixture of acetic anhydride–HOAc–THF (2:1:3). Treatment of that same benzylidene protected disaccharide intermediate **91** with HOAc–H_2_O (4:1) at 70 °C gave the corresponding diol **93** with the Dts group completely unaltered by the conditions of acid hydrolysis. Next, glycosylation of **93** with **83** in the presence of TMSOTf gave trisaccharide **94**, which was treated further with zinc in the manner already stated to give the core 4 building block **95** in 65% overall yield.

**Scheme 16:**
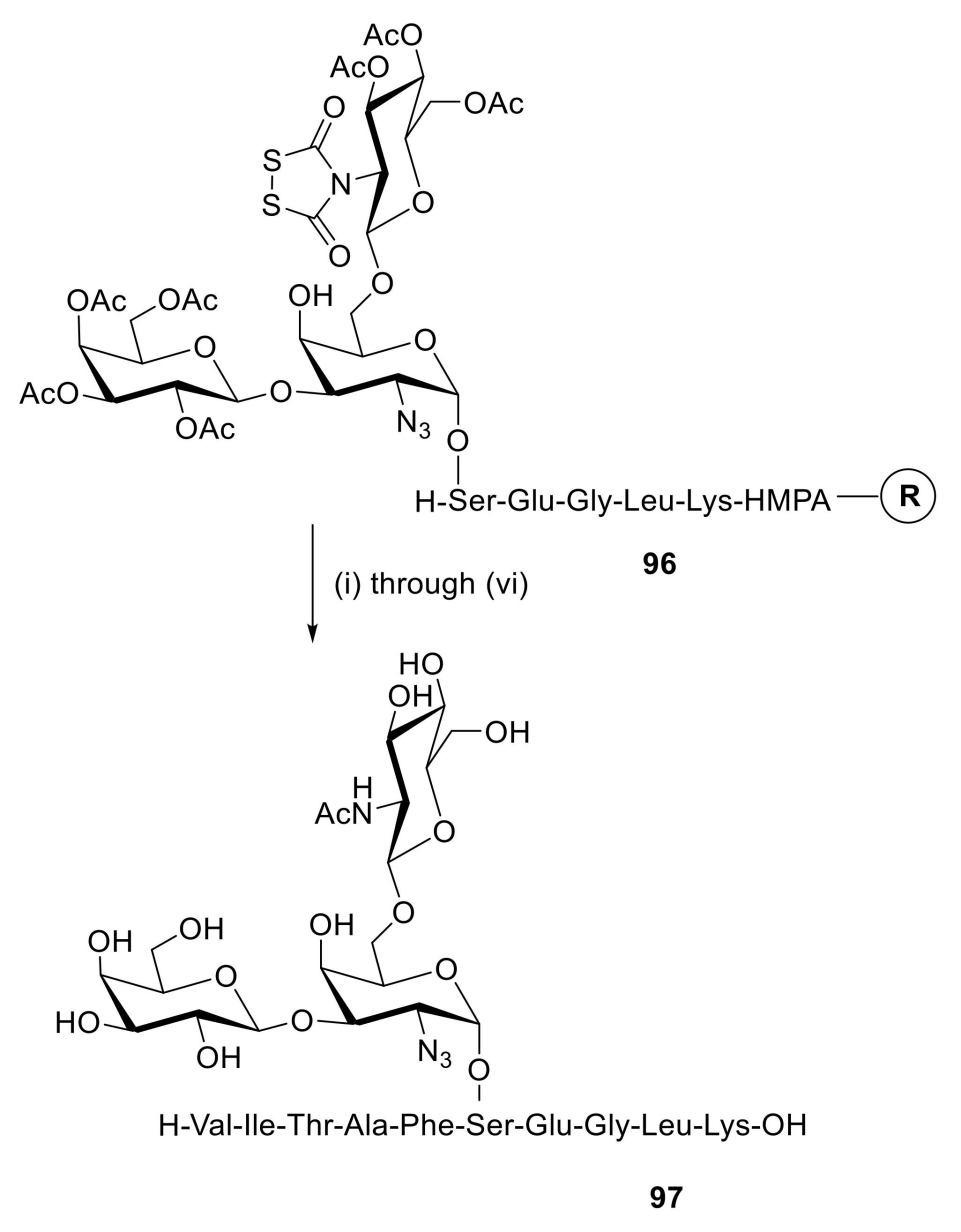
Solid-phase removal of the Dts function in the presence of an azide group, taken from Meinjohanns et al. [146]. (i) PDT (0.2 m)–DIEA (0.3 m) in CH_2_Cl_2_; (ii) Ac_2_O–DMF (1:4); (iii) stepwise Fmoc SPPS; (iv) piperidine–DMF (1:4); (v) TFA–H_2_O (19:1); (vi) 1% NaOMe in MeOH (pH 8.5).

In order to investigate the role of glycosylations on T-cell response [146], the Copenhagen group used several of the above Dts-protected building blocks in the synthesis of a series of analogues of the peptide fragment of CBA/J mouse hemoglobin Hb (67–76), H-Val-Ile-Thr-Ala-Phe-Asn-Glu-Gly-Leu-Lys-OH. One aspect of their study was to develop selective conditions for solution or solid-phase Dts removal in the presence of azido groups in solution and on solid phase. This goal was met by two consecutive 10-min applications of propane-1,3-dithiol (PDT ≡ HSCH_2_CH_2_CH_2_SH) (0.2 m)–DIEA (0.3 m) in CH_2_Cl_2_ (Scheme 16). Additional steps were straightforward, and the desired glycopeptide **97** was obtained from protected glycopeptide-resin **96** in 76% yield after HPLC purification. In an alternative approach, *N*-Dts and azido groups were efficiently reduced, at the same time, by treatment of a glycopeptide-resin with DTT (0.2 m)–DIEA (0.1 mm) in CH_2_Cl_2_.

## 10. Additional Results in Glycopeptide Synthesis (Minneapolis)^34^

**Figure 3:**
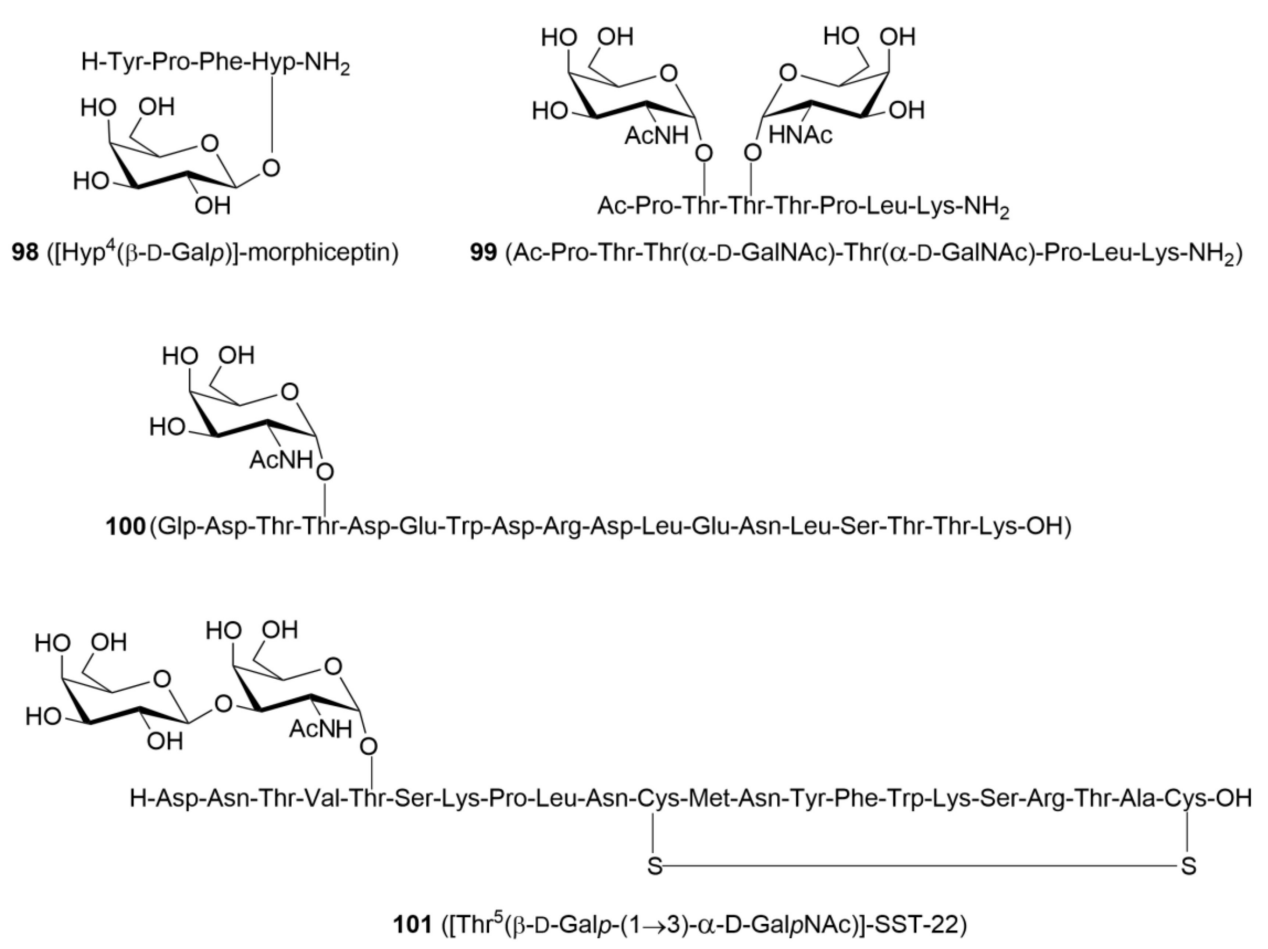
Representative glycopeptides prepared under mild conditions by Barany and collaborators, using Fmoc solid-phase peptide synthesis, along with suitable protected building blocks, handle–resin supports, and orthogonal cleavage protocols. See text for further details, including additional variants made, and literature citations.

Beyond our work using the Dts function to protect the 2-amino position of glucosamine, as was summarized in the preceding section of this perspective, our Minneapolis laboratory has had a decades-long interest in glycopeptide synthesis (Figure 3). Some of this was fueled by Fernando Albericio from the University of Barcelona and (separately) Copenhagen-trained Knud Jensen^35^, both of whom have already been introduced to readers, and significant contributions were from Eduard Bardají, then at the Centro de Investigacion y Desarrollo, Consejo Superior de Investigacion y Ciencia (CID-CSIC) in Barcelona, Spain; Jan Tejbrant, a postdoctoral fellow from Stockholm, Sweden; and Lin Chen, an organic chemist originally from China who, under GB´s tutelage (both graduate and postdoctoral work) mastered peptide chemistry and continued his career making commercially significant peptides with Roche and Corden Pharma in Boulder, Colorado. In the new millennium, it was a privilege for GB to conduct separate rewarding collaborations with David Live and with David Hamilton, with the majority of the synthetic work, as well as assimilation of the literature, was conducted by Mian Liu, a postdoctoral fellow from China [156,157,158,159,160]. An early success was the solid-phase synthesis of morphiceptin analogues (e.g., **98** in Figure 3 with galactose; also carried out with glucose) that were *C*-terminal peptide amides [161]. Published ahead of our full paper on PAL (though a communication [57] was already out), this contribution showed for the first time how milder Fmoc/tBu/PAL chemistry (Scheme 3) could be used to make glycopeptides. Standard Boc/benzyl chemistry (Scheme 1) or its variations are unsuccessful for this purpose, due to the fact that HF breaks *O*-glycosidic linkages.

The required anomerically pure glycosylated building blocks, with *O*-acetyl groups on all free hydroxyls of the sugar, were prepared by literature methods. Once the linear protected glycotetrapeptide was assembled on PAL-amino polystyrene by Fmoc SPPS, quantitative *on-resin* deacetylation with hydrazine–MeOH (4:1), for 2 h, was followed by treatment with TFA–CH_2_Cl_2_ (7:3) to release glycopeptide from the support. Initial yields and purities were excellent, and efficient reversed-phase HPLC gave highly pure products in good overall yields.

The Copenhagen work on mucin-like glycopeptides has been described earlier in this perspective (Scheme 15 and accompanying discussion). In a program eventually geared to studying conformational preferences by ^1^H NMR and understanding how these relate to biological activity, we had occasion to prepare a suite of seven partially glycosylated heptapeptides (e.g., **99** in Figure 3) and systematically evaluate several synthetic tactics [158]. *N*^α^-Fmoc-*O*-(3,4,6-tri-*O*-acetyl-2-azido-2-deoxy-α-d-galactopyranosyl)-l-threonine pentafluorophenyl ester [Fmoc-l-Thr(Ac_3_-α-d-GalN_3_)-OPfp] was used as a building block that coupled efficiently when used in a relatively low molar excess, i.e., 1.5 equiv, with DMF as the solvent.

For conversion of the azido group to the *N*-acetyl function, direct treatment with thioacetic acid was preferred over a two-step procedure involving reduction with dithiothreitol (DTT) followed by *N*-acetylation. Effective *O*-deacetylation of these glycopeptides was achieved by treatment with sodium methoxide (10–15 mm; 5 equiv) in methanol. On-resin deacetylation techniques were also examined, using sodium methoxide (6–10 mm) in DMF–methanol (17:3), or hydrazine (70 mm) in methanol. The more convenient on-resin technique in DMF–methanol gave yields similar to solution conditions, and we recommend it for future solid-phase glycopeptide synthetic studies.

In another project of interest, we set the stage for using solid-phase glycopeptide synthesis to address an interesting biological question. An 18-residue sequence appears at the *N*-terminus of the rat epididymal **c**ysteine-**ri**ch **s**ecretory **p**rotein (Crisp-1) that is important in the fertilization process. Stepwise Fmogc SPPS using a convergent strategy and standard side-chain protecting groups was applied to prepare the parent sequence, plus two possible α-*O*-linked T_N_ antigen-containing glycopeptides with a Thr(α-d-GalNAc) residue in place of either Thr^3^ or Thr^4^ [the last of these is structure **100** in Figure 3].

Starting with *C*-terminal Lys(Boc) anchored as a PAB ester to a CLEAR support^36^, the first 14 residues were assembled on an automated continuous-flow instrument. Fmoc removal was achieved with piperidine–NMP (1:4) for 30 min, and couplings (4 equiv of Fmoc-amino acids) were mediated by 2-(1*H*-6-chlorobenzotriazole-1-yl)-1,1,3,3-tetramethyluronium hexafluorophosphate (HCTU)–HOBt–DIEA (1:1:2) in DMF. The final four residues were incorporated manually by the same chemistries, with the Fmoc-Thr(Ac_3_-α-d-GalNAc)-OH building block (1.5 equiv) being coupled for 4 h to compensate for the lower excess. Upon completion of solid-phase chain assembly, peptides were cleaved from the support, and amino acid side-chains were simultaneously restored, upon treatment with Reagent K, TFA–thioanisole–1,2-ethanedithiol–phenol–H_2_O (82.5:5:2.5:5:5), at 25 °C for 2 h.

During automated chain assembly, two deletion peptides [des-Asp^2^ and des-Thr(Ac_3_-α-d-GalNAc)] and one terminated peptide [*N*-acetylated 14-mer] arose, as did several peptides in which aspartimide formation had occurred at each of the four possible positions in the sequence. These by-products totaled ~20% of the desired product; they were recognized by HPLC and ESI-MS and removed during the intermediate purifications. Final products, obtained in 15–21% overall yields, were characterized by HPLC purities and ESI-MS. Future biological evaluation could elucidate the nature and locus of sugar modification of Crisp-1, and provide insight as to why Crisp-1 protein E binds sperm irreversibly, in contrast to protein D that lacks a sugar near the *N*-terminus and only binds sperm loosely.

Catfish somatostatin (**101** in Figure 3), which features a glycoform-containing d-galactosamine and d-galactose *O*-glycosidically linked to threonine, along with an intramolecular disulfide bridge, was a biologically interesting target to showcase some of our synthetic know-how [162]. The linear sequence was assembled smoothly by Fmoc SPPS, starting with an Fmoc-Cys(Trt)-OPAB-PEG–PS support. The building block for incorporating the sugar linked to threonine was *N*^α^-Fmoc-Thr(Ac_4_-β-d-Gal(1→3)-Ac_2_-α-d-Gal(NAc)-OH (a non-glycosylated control was made in parallel, along with a variant in which Thr was linked to a monosaccharide). Acidolytic deprotection/cleavage of the full-length (22 amino acid residues) protected peptide–resin with appropriate TFA/scavenger cocktail gave the corresponding acetyl-protected glycopeptide with free sulfhydryl functions. Deacetylation by methanolysis in the presence of catalytic sodium methoxide was followed by mild oxidation at pH 7, mediated by *N*^α^-dithiasuccinoyl (Dts)-glycine (**20**, R = H). This last step was a demonstration, for a complex target peptide, of a mild method for intramolecular disulfide cyclization that in effect runs the mechanism for thiolytic removal of Dts (Scheme 7) “backwards” and favors monomeric products [115]. Preparative HPLC gave pure product in an overall 4% isolated yield, which is very respectable for a molecule of this complexity.

## 11. Invention of Further PEG-Based Supports for SPPS and Other Applications

By the mid-1990s, based on our initial work with PEG–PS, Rapp’s parallel work with TentaGel, and Meldal’s innovative work with PEGA, a consensus had emerged that there were concrete advantages to carrying out SPPS on resin supports that had a substantial hydrophilic character so as to be more compatible with the growing (solvated) peptide chains. These new families of supports were deemed to be improvements over the cross-linked polystyrene resins that had been standard since Merrifield’s pioneering work in the 1960s and continuing through most of the several decades that followed.

Bonus properties of PEG-based supports were amenability to continuous-flow modes of SPPS and compatibility with, on the one hand, reagents and reaction conditions used by organic chemists and, on the other hand, the aqueous milieus needed for enzymatic reactions and/or biological binding assays. In these regards, PEG-based supports were central to the burgeoning developments of *combinatorial chemistry* spurred by Árpád Furka and Kit Lam’s one bead-one peptide (split pool) concept that was announced in the early 1990s [163,164,165,166].

Based on the concepts and the tangible results just summarized, there was a considerable impetus to invent further PEG-based supports, and the present portion of this perspective covers several of these, with an emphasis on studies carried out in Copenhagen and in Minneapolis.

Maria Kempe carried out her doctoral studies on molecular imprinting with Klaus Mosbach at Lund University, in the twin cities of Lund/Malmö, Sweden,^37^ and it was GB’s good fortune to convince her to join the Minneapolis laboratory, where she was supported in part by a Hans Werthén postdoctoral fellowship (1994–1996). With considerable creativity and experimental skill, Kempe applied her experience with polymerization to invent a new class of supports termed “CLEAR”, an acronym for **C**ross-**L**inked **E**thoxylate **A**crylate **R**esin [167]. As described below, these materials had superb properties for SPPS, stressing the beneficial contributions from their PEG-like milieu and belying the conventional wisdom that supports for SPPS needed to have the minimum level of crosslinking that still maintained mechanical stability. 

**Figure 4:**
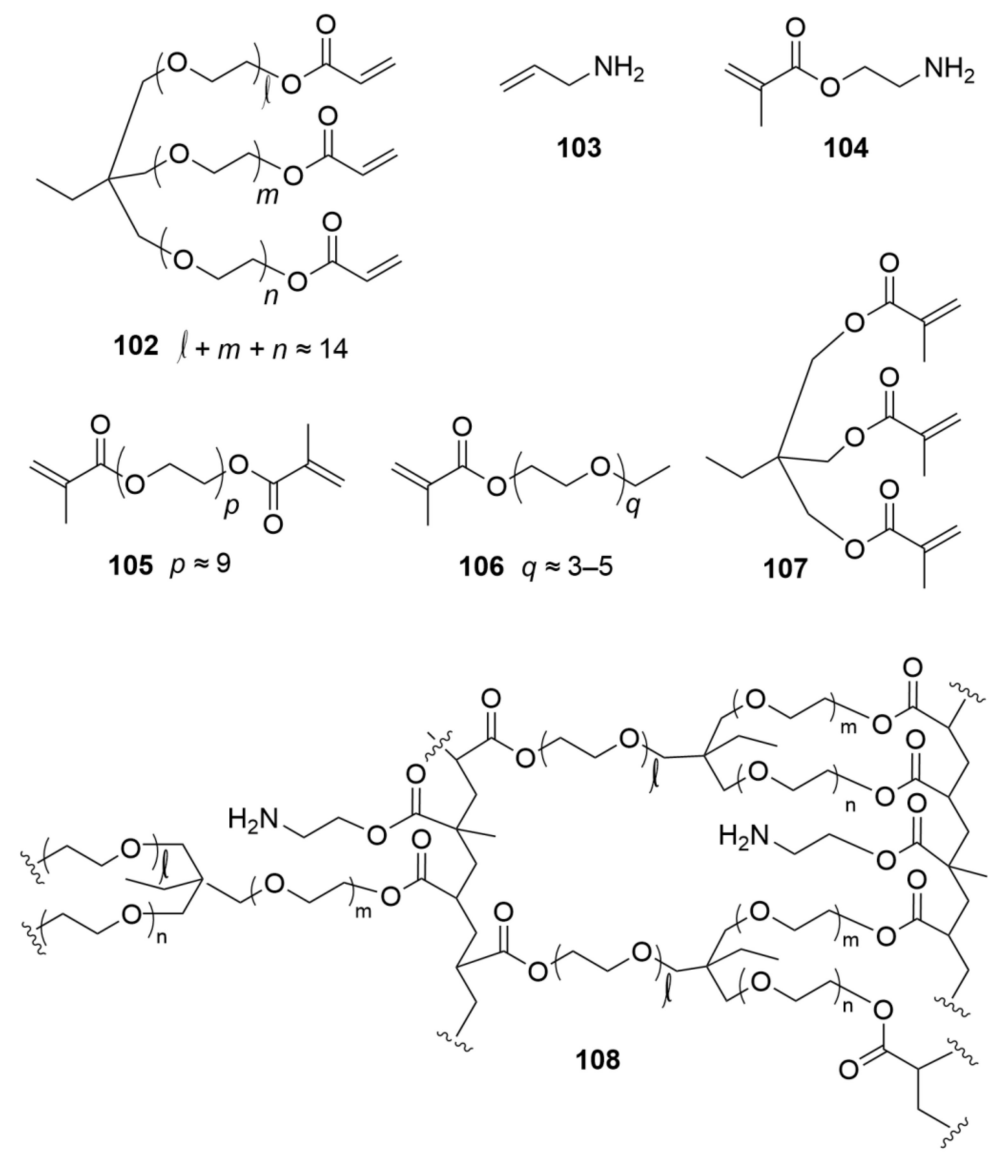
Monomers (**102**–**107**) that, in various combinations, co-polymerize to give rise to CLEAR (**C**ross-**L**inked **E**thoxylate **A**crylate **R**esin) supports (e.g., **108**, which is specifically derived from **102** and **104**). Modified from Kempe and Barany [167] and Barany et al. [125].

**Figure 5:**
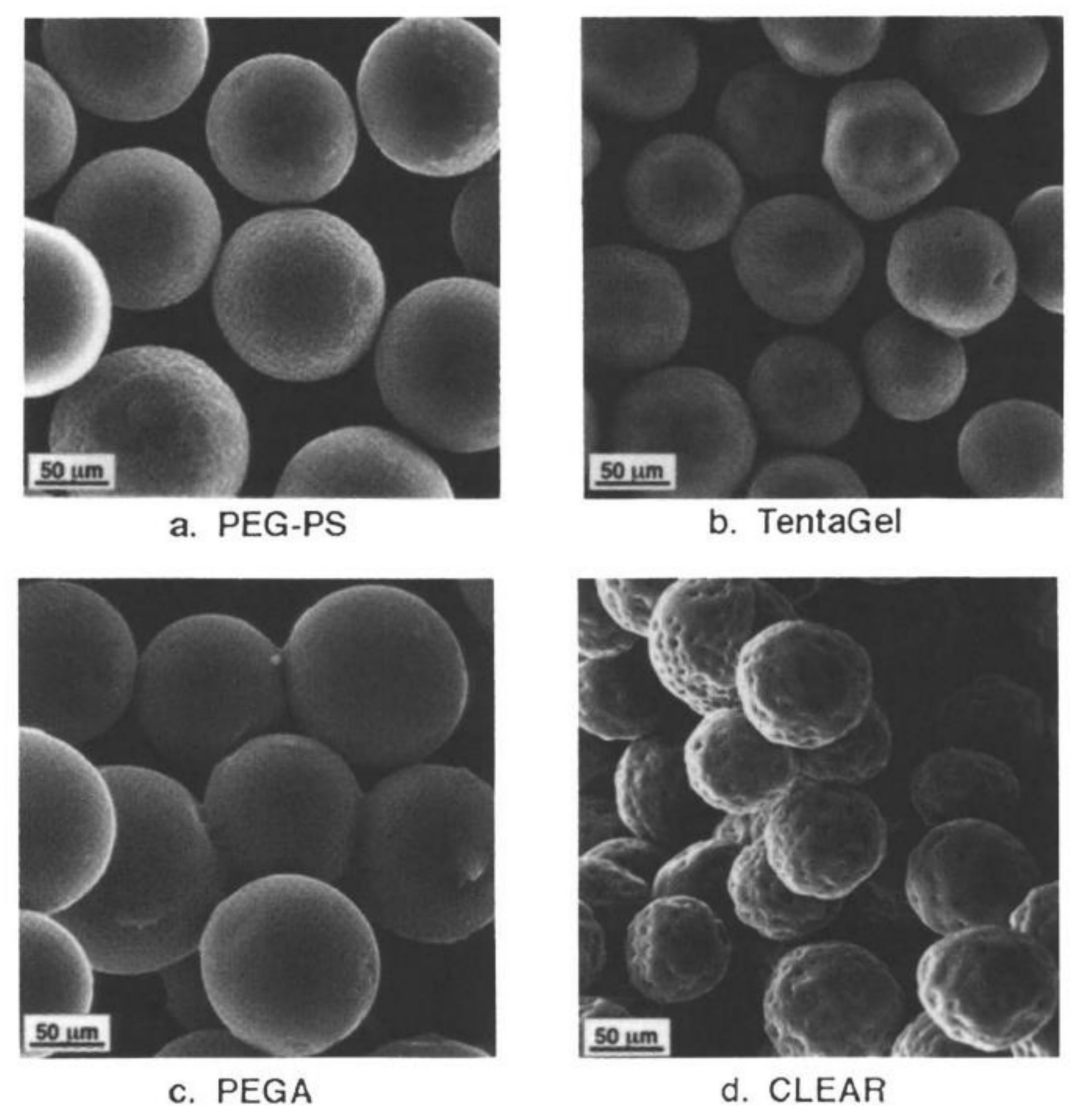
Scanning electron micrographs (accelerating voltage: 10 kV) showing the shape and texture for each of (**a**) PEG–PS, (**b**) TentaGel, (**c**) PEGA, and (**d**) CLEAR-IV. Reproduced from ref. [125].

The CLEAR family of supports (**108**) were prepared by radical copolymerization, either in the bulk or suspension mode, of the branched cross-linker trimethylolpropane ethoxylate (14/3 EO/OH) triacrylate (**102**) with *either* allylamine (**103**) or 2-aminoethyl methacrylate·HCl (**104**), along with (*optionally*) one or more of poly(ethylene glycol-400) dimethacrylate (**105**), poly(ethylene glycol) ethyl ether methacrylate (**106**), or trimethylolpropane trimethacrylate (**107**) (Figure 4). The resultant highly cross-linked copolymers, when made by bulk procedures, were ground and sieved to give particles. When the optimized suspension polymerization procedure was followed, **108** was obtained as highly cross-linked spherical beaded supports (Figure 5d).

CLEAR polymeric supports showed excellent swelling properties in an unusually broad range of solvents, including water, alcohols, tetrahydrofuran, acetonitrile, CH_2_Cl_2_, and *N*,*N*-dimethylformamide. To demonstrate their usefulness for Fmoc SPPS, CLEAR supports were used to build a series of challenging peptides such as acyl carrier protein (65–74), retro-acyl carrier protein (74–65), and the 17-peptide human gastrin–I, in both batchwise and continuous-flow modes [167].

Meanwhile, across the pond, Meldal was concerned that PEGA is not sufficiently stable towards harsh chemicals and heating, largely due to its amide bonds. Consequently, in collaboration with postdoctoral fellow Manat Renil at Carlsberg, Meldal reported two PEG-based resins, namely polyoxyethylene–polystyrene (POEPS) **114** and polyoxyethylene–polyoxypropylene (POEPOP) **115**, where all of the amide linkages present in PEGA were replaced by ether bonds [168]. As a follow-up, Meldal in collaboration with graduate student Jens Buchardt, developed yet another support, termed “POEPS-3 (**116**) resin” [169]. This interspersed three methylene groups between the PEG moiety and the polystyrene backbone.

**Figure 6:**
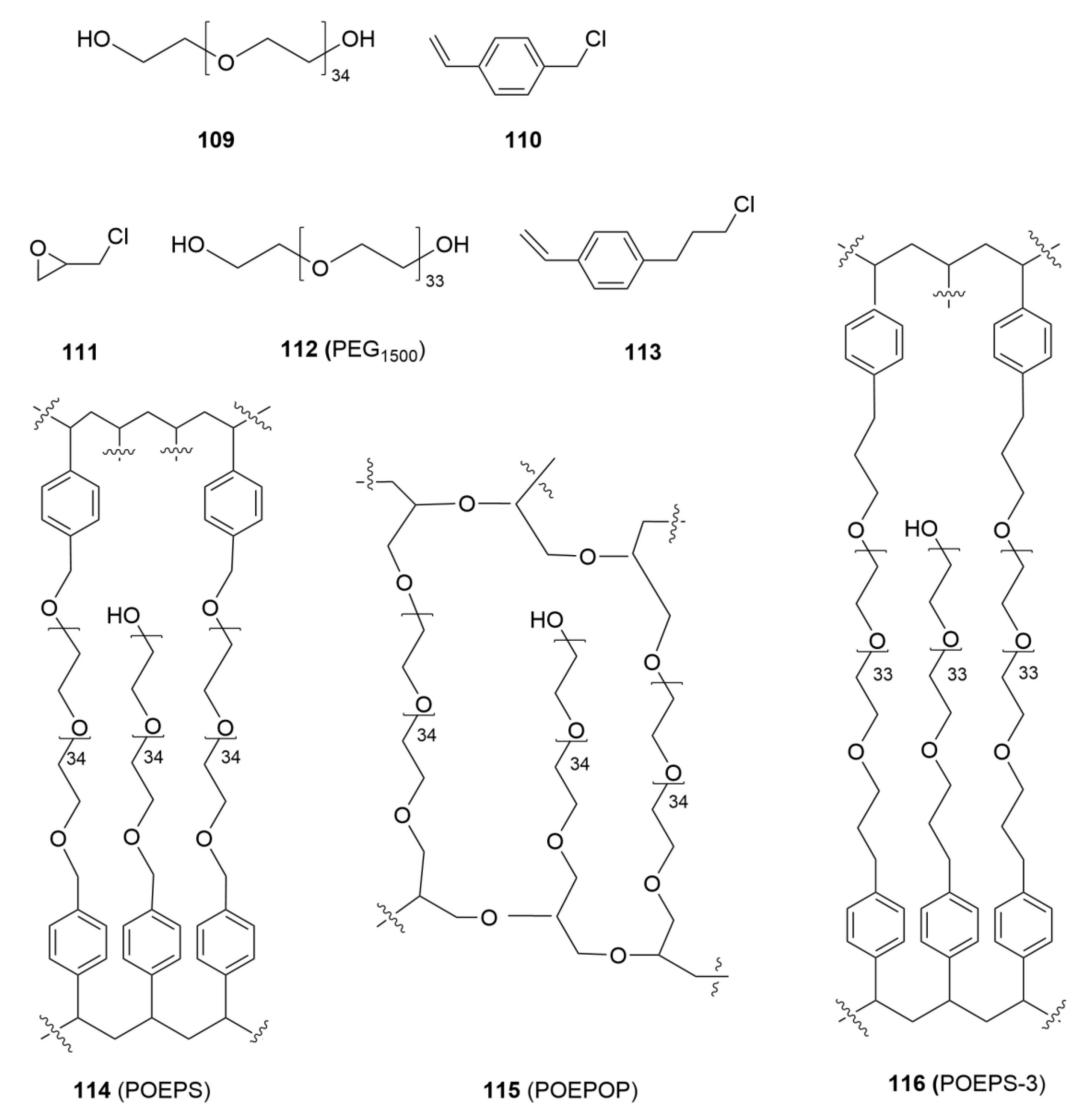
Monomers (**109**–**113**) that co-polymerize to give rise to POEPS (**114**), POEPOP (**115**) and POEPS-3 (**116**) supports. Modified from refs. [168,169].

The Copenhagen team hypothesized that these structural redesigns (Figure 6) would make it possible to perform a wider range of organic reactions, e.g., those involving generation—as reactive intermediates or as stable reagents—of carbocations, carbanions, and/or carbenes. Nevertheless, it was required that these materials would still be compatible with biochemical reactions in aqueous media, as is the case with PEGA.

The PEG resins **114** and **115** were synthesized by co-polymerization of bifunctional PEG-diol (**109**) of average molecular weight 1500 with 4-vinylbenzyl chloride (**110**) and epichlorohydrin (**111**) respectively. The pentapeptide H-Gly-Phe-Ser-Phe-Gly-NH_2_ was synthesized on both resins, in 93% and 86% yield, respectively, by Fmoc SPPS using Fmoc-AA-OPfp (2.5 equiv) building blocks in the presence of Dhbt-OH, which latter served as an indicator for completion of the reaction (yellow color gone within 15 min).

As an impetus for developing PEG resin **116**, Meldal noted that in POEPS (**114**) [and, for that matter, in TentaGel as well], PEG is attached to a polystyrene backbone *via* a benzylic ether bond, which could be cleaved by catalytic hydrogenolysis or upon exposure to Lewis acids. The new variation was prepared by inverse suspension radical polymerization of the appropriate macromonomers consisting of PEG-1500 partially derivatized with 4-(3-chloropropyl)-styrene (**113**).

PEOPS-3 resin showed good swelling in DMF, H_2_O, and CH_2_Cl_2_. To evaluate their relative stabilities, POEPS (**114**) and POEPS-3 (**116**) were both treated with trimethylsilyl trifluoromethanesulfonate (2 equiv) and Ac_2_O (45 equiv) in CH_2_Cl_2_ at 25 °C, which are conditions often used to cleave benzyl groups in carbohydrate chemistry. Resin **114** was completely dissolved after 10 min, while resin **116** appeared unchanged after 50 min.

**Figure 7:**
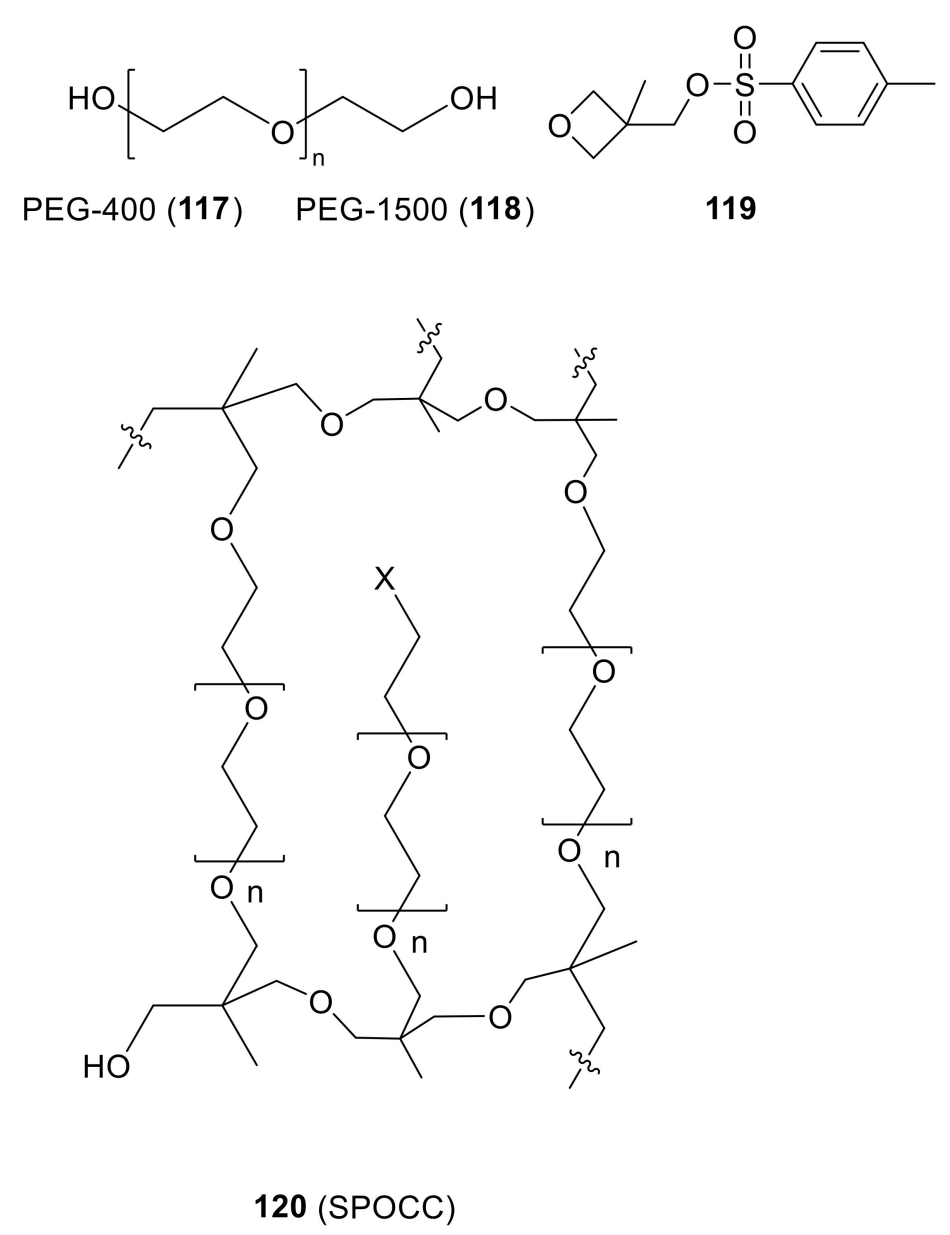
Monomers (**117**, **118** and **119**) that co-polymerize to give rise to SPOCC supports (**120**). For **117**, n ≈ 8, and for **118**, n ≈ 33. The free hydroxyl groups of the SPOCC supports (**120** with X = OH) can be readily converted to amino, thiol, or bromo functions [170].

With a new millennium on the horizon (see next section of this perspective), Meldal and coworkers reported on the natural culmination of their research on PEG-based supports [170]. Thus, they announced the design, preparation, and properties of the so-called “**s**uper**p**ermeable **o**rganic **c**ombinatorial **c**hemistry” (SPOCC) resin (**120**), which is a cross-linked polymer that is comprised exclusively of primary ether and alcohol functionalities (Figure 7).

To prepare SPOCC resins, a suitable-sized PEG chain (**117** or **118**) was treated with potassium bis(trimethylsilyl)amide (KHMDS), followed by 3-methyl-3-[[(4-tolylsulfonyl)oxy]methyl]oxetane (**119**), and polymerized further with boron trifluoride diethyl etherate (BF_3_**^.^**Et_2_O) as a Lewis acid catalyst. The product materials were obtained in beaded form.

The SPOCC resin (**120**) was shown to be inert to strongly acidic (e.g., anhydrous HF; 35% HBr in HOAc) and basic conditions (e.g., 20 equiv of *n*-butyllithium), and to most reaction conditions used in organic reactions.

**Scheme 17:**
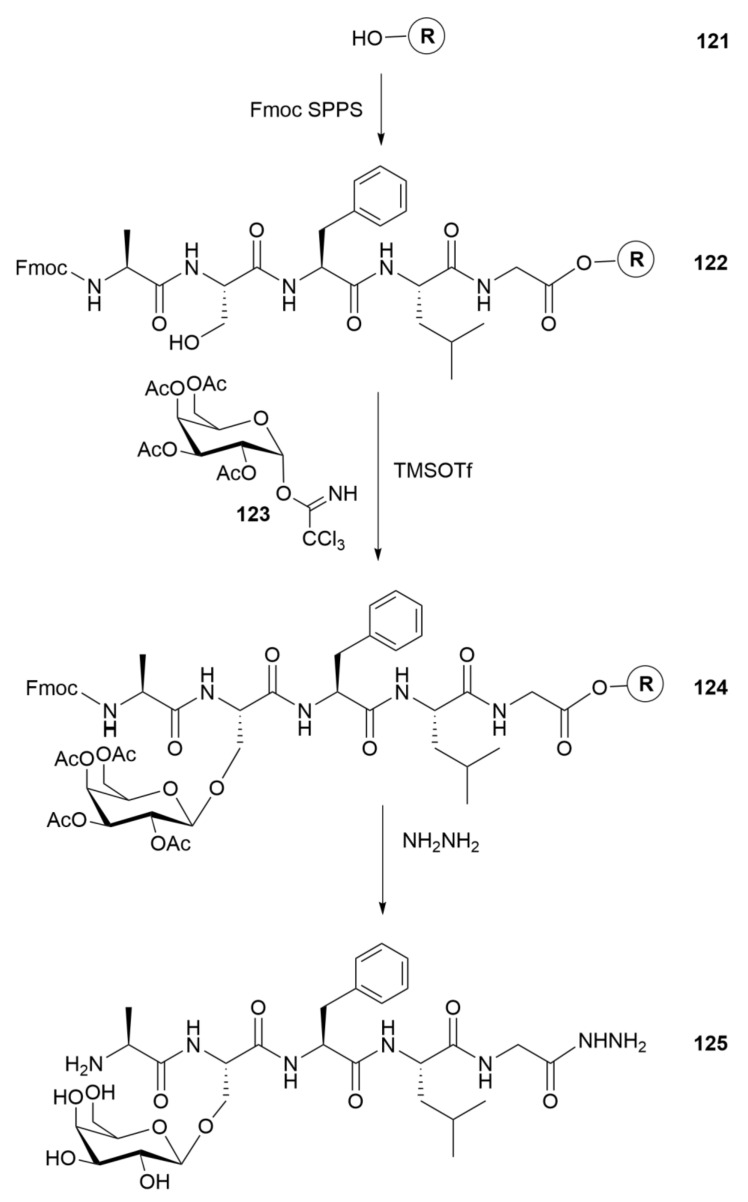
Glycopeptide synthesis on SPOCC-400 [170]. The pentapeptide sequence Ala-Ser-Phe-Leu-Gly, with side-chain unprotected Ser, was assembled by stepwise Fmoc SPPS [couplings mediated by TBTU/NEM; deprotections with piperidine–DMF (1:4)]. The on-resin glycosylation step used tetra-*O*-acetyl-α-d-galactopyranosyl trichloroacetimidate (**123**) in the presence of TMSOTf as promoter. Treatment of peptide-resin **124** with hydrazine simultaneously removed the *N*-Fmoc and the acetyl protecting groups, while releasing the final peptide product **125** as its *C*-terminal peptide hydrazide.

As Meldal had hoped, SPOCC resin proved to be useful for SPPS and a wide range of organic reactions in water and organic solvents. Nucleophilic examples of the latter included the synthesis of peptide isosteres by reaction of *N*-terminal aldehydes with ylides in Wittig-type and Horner–Wadsworth–Emmons-type reactions. Electrophilic reactions on SPOCC included solid-phase glycosylation for the synthesis of glycopeptides (Scheme 17). As a final demonstration of the versatility of SPOCC resin, the Copenhagen team showed that the 27 kDa protease subtilisin BNP´ could access the interior of an appropriate peptide–resin and cleave a decapeptide substrate.

**Figure 8:**
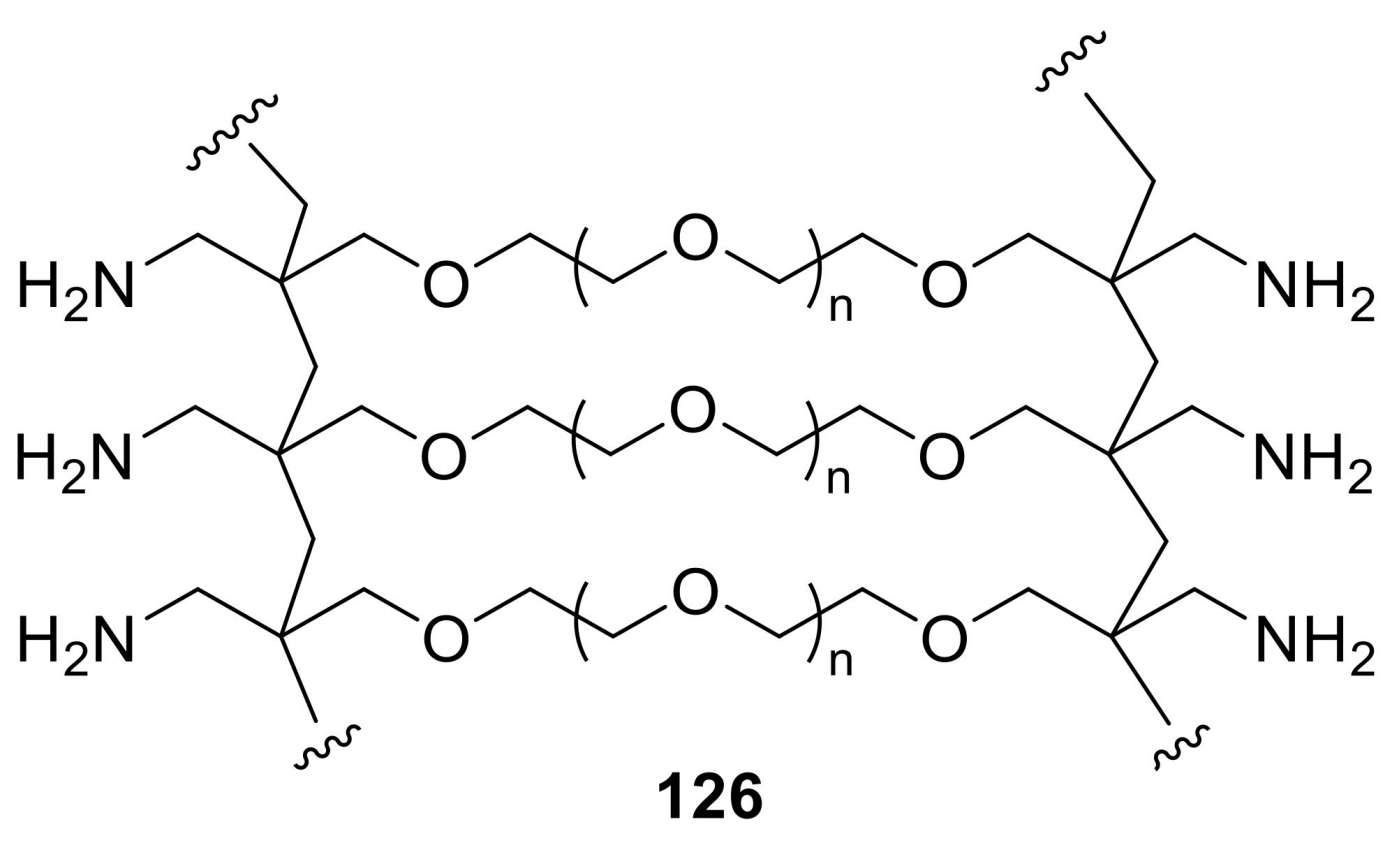
General structure of ChemMatrix resin, showing its high PEG content [171,172,173,174,175]. The experimental details about the synthesis of these materials are proprietary, so the authors of this perspective are unable to make a direct comparison to other PEG-based supports covered herein.

Independent of the work of Meldal on SPOCC, as just summarized, a small Canadian company named PCAS BioMatrix made and marketed (2005–2022) a proprietary PEG-based support that they termed ChemMatrix (Figure 8). The company’s co-founder and President, Simon Côté, was the sole inventor listed on a 2005 patent for this material, but all publicly available refereed publications on this material have Fernando Albericio (also a co-inventor of PEG–PS) and Judit Tulla-Puche, both having been trained in Minneapolis^38^ but affiliated at the time with the University of Barcelona, as corresponding authors [171,172,173,174,175].

Thus, ChemMatrix contains only primary ether bonds, and swells well in a wide range of solvents, including CH_3_CN, DMSO and MeOH. The Fmoc SPPS synthesis of several challenging peptides, e.g., β-amyloid (1–42) peptide, the 28-residue peptide thymosin α1, analogs of gene-related calcitonin peptide (1–37) and chemokine RANTES (1–68), has been documented, in some cases exploiting pseudoproline dipeptide building blocks to navigate through difficult couplings [174,176,177]. ChemMatrix has also been reported to provide favorable results for PNA synthesis [178] and for combinatorial chemistry [175].

## 12. Dts Chemistry in the Service of PNA Synthesis

When introduced in a landmark 1991 *Science* paper [179] by Peter Nielsen, Michael Egholm, Ole Buchardt, and Rolf Berg, from the University of Copenhagen, Denmark, peptide nucleic acids (PNA) captured the imaginations of many scientists (ourselves included)^39^ working at the interface of bioorganic chemistry and molecular biology. The overall concept was disarmingly simple, yet elegant: assemble a poly(pseudopeptide) backbone with the various nucleobases found in DNA now presenting as side-chains, while avoiding the charge and chirality of oligonucleotides. It was hoped that well-defined PNA sequences could hybridize with each other or with single-stranded DNA or RNA partners, in a highly specific manner dictated by the classical Watson–Crick complementarity of the bases. Indeed, this plan came to fruition in the laboratory, as described in the foundational studies from Copenhagen [179,181,182,183,184,185,186] and has been summarized most recently in a review by Albericio and coworkers [187].

In our eyes, PNA presented additional opportunities: (a) the entire tool kit of peptide synthesis could be harnessed to build up oligomers required for the molecular biology applications; (b) since PNA monomers are achiral, concerns about racemization are moot, and stronger levels of activation could be pursued; (c) PNA might lend itself to segment condensation approaches, with trimer or tetramer building blocks that could be coupled to create longer oligomers^40^^,^^41^; and (d) the poly(2-aminoethylglycyl) backbone of PNA precludes hydantoin formation (compare to Scheme 11) of the kind that was so problematic when the Dts protecting group was used on more difficult sequences in stepwise SPPS—could this be a “second chance” for Dts?

Knud Jensen, whom readers of this perspective will recognize as having been Meldal’s first Carlsberg Ph.D. student and carried out his postdoctoral studies in Minneapolis; and Eduard Bardají, a visiting Professor from University of Girona (Spain), together jump-started a project to study the use of Dts for PNA synthesis (the pilot studies used only the side-chain of thymine (T)). This effort was continued by Bardají’s Girona faculty colleague, Marta Planas, who spent two full calendar years (1997 and 1998) in GB‘s laboratory under the auspices of fellowships first, from NATO, and second from the Ministerio de Educación y Cultura (MEC) of Spain. Our findings, with a complete set of protected PNA monomers, were published in 1999 in *J. Org. Chem.* [192].

**Scheme 18:**
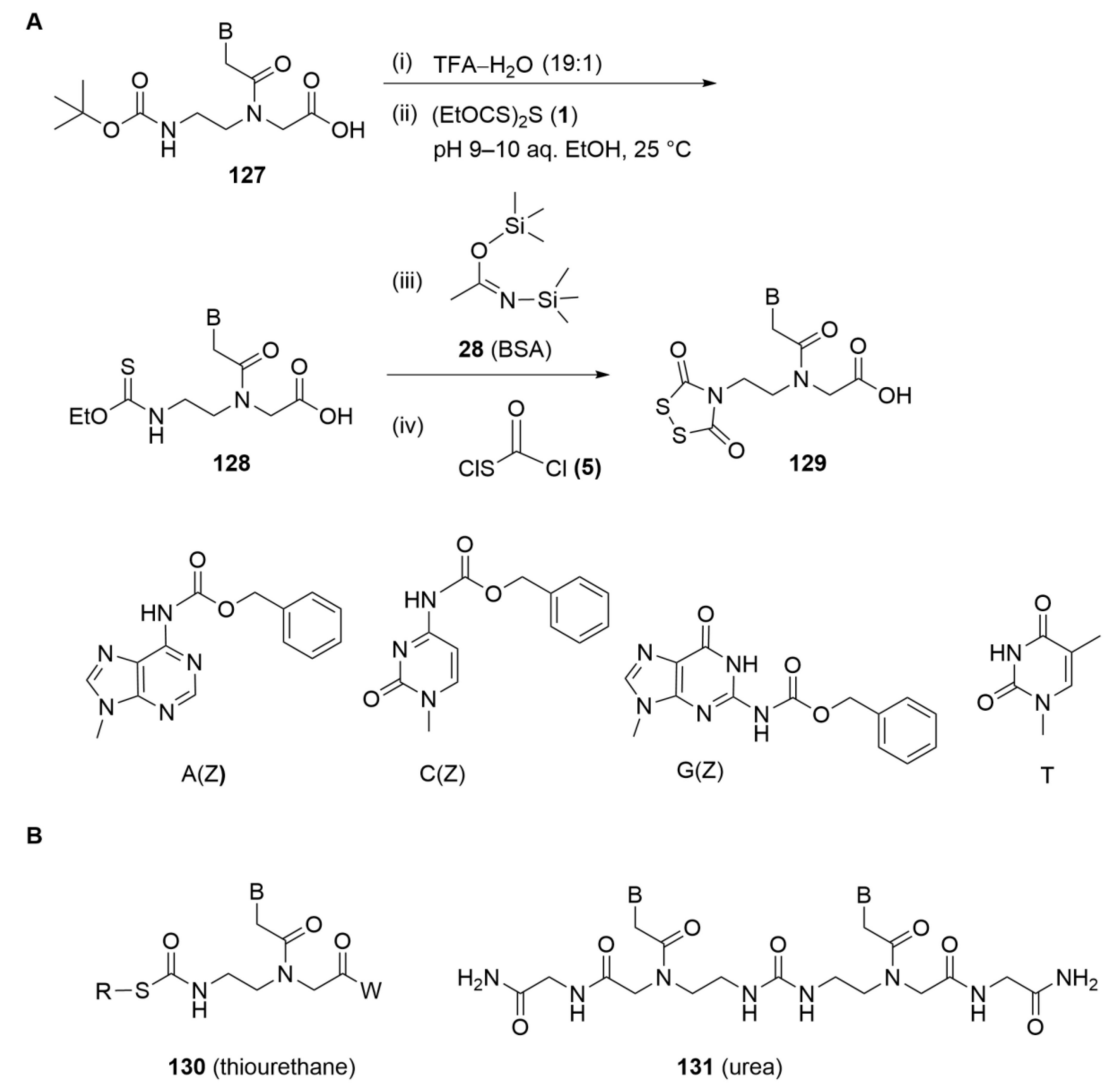
(**A**) Preparation of *N*^ω^-Dts-protected building blocks for PNA synthesis. (**B**) Structures of some by-products encountered under sub-optimal conditions. For more information, see Planas et al. [192].

In brief, previously precedented protocols [i.e., Scheme 4A, for Dts creation; Scheme 7 for Dts removal] were readily adapted to PNA (Scheme 18), although there were a few “traps” along the way. To begin, commercially available *N*^ω^-Boc/side-chain benzyloxycarbonyl (Z)-protected PNA monomers (**127**) were converted to the corresponding derivatives (**129**) protected by the *N*^ω^-dithiasuccinoyl (Dts) function by the following sequence: (i) acidolytic removal of Boc; (ii) treatment with bis(ethoxythiocarbonyl)sulfide (**1**) to give the *N*^ω^-ethoxythiocarbonyl (Etc) derivatives (**128**); (iii) *in situ* silylation at the α-carboxyl; (iv) ZWK reaction with (chlorocarbonyl)sulfenyl chloride (**5**). Some of these steps required special care in the experimental conditions, e.g., the Etc derivatives had to be used freshly to avoid spontaneous rearrangement (partial) to the deleterious Emc isomers [**7** in Scheme 4B; **130** (W = OH) in Scheme 18B], and the ZWK reaction had to be carried out at 0 °C in order to avoid Tda formation [**13** in Scheme 4B]. Gratifyingly, there did not appear to be any Eoc by-products formed [**8** in Scheme 4B]. All told, the net yields of pure monomers were 71–78%.

Conditions in the solid-phase mode for thiolytic removal of the Dts group, and for coupling of protected monomers, were then studied in extensive model studies, and optimized accordingly. One concern was that certain thiols, when applied to the thiolytic removal of the *N*^ω^-Dts (compare to Scheme 7), engaged (in part) in a nucleophilic displacement at the Dts carbonyl instead, providing unwelcome thiourethane side products (**130** in Scheme 18B). Fortunately, we were able to identify appropriate thiols, along with conditions for their effective use, so that Dts removal from PNA occurred quickly, quantitatively, and *without* formation of thiourethanes that would serve as chain terminators.

Another issue that we noticed and addressed was urea formation (**131** in Scheme 18B). This side reaction occurred during the on-resin thiolytic deprotection step, when a liberated *N*^ω^-amine would attack the Dts carbonyl from an adjoining PNA chain, in an intermolecular process. The remedy, however, was exceedingly simple, i.e., simply reducing the overall loading of growing PNA chains on the support.

Based on the findings just cited, a protocol featuring (i) Dts removal with dithiothreitol (DTT) (0.5 m) in acetic acid (HOAc) (0.5 m)–CH_2_Cl_2_ (2 + 8 min); (ii) short neutralization with DIEA–CH_2_Cl_2_ (1:19, 1 + 2 min); and (iii) coupling mediated by HBTU–DIEA (3:1) in NMP (3 h) was applied to the solid-phase synthesis of Dts-T_4_-Gly-NH_2_, Dts-G(Z)-G(Z)-T-A(Z)-Gly-NH_2_, Dts-A(Z)-T-C(Z)-G(Z)-Gly-NH_2_, and Dts-G(Z)-C(Z)-A(Z)-T-Gly-NH_2_. The indicated protected PNA derivatives were released from the PAL support with TFA-H_2_O (19:1) for 2 h and their structures were verified by mass spectrometry.

## 13. Peptides for the New Millennium: Minneapolis 1999

Since 1968, the American Peptide Society has held biennial meetings at locations throughout North America. In 1999, it was our privilege to host the 16^th^ such Symposium at the Convention Center in downtown Minneapolis, from 26 June to 1 July. GB and Gregg B. Fields^42^ were the co-Chairs, and there were approximately 1200 participants from some three dozen different countries. Morten Meldal gave an invited lecture on Monday of that week, entitled “SPOCC Resins: Polar and Chemically Inert Resins for Organic Synthesis and Enzyme Library Assays”, which foreshadowed the momentous discovery that would be reported at the 17^th^ Symposium just two years hence. The lecture, along with several posters from the Copenhagen group on various aspects of combinatorial chemistry, was published in the Symposium proceedings [193]. Morten was accompanied by his coworker and future second wife Phaedria Marie St. Hilaire.

**Figure 9:**
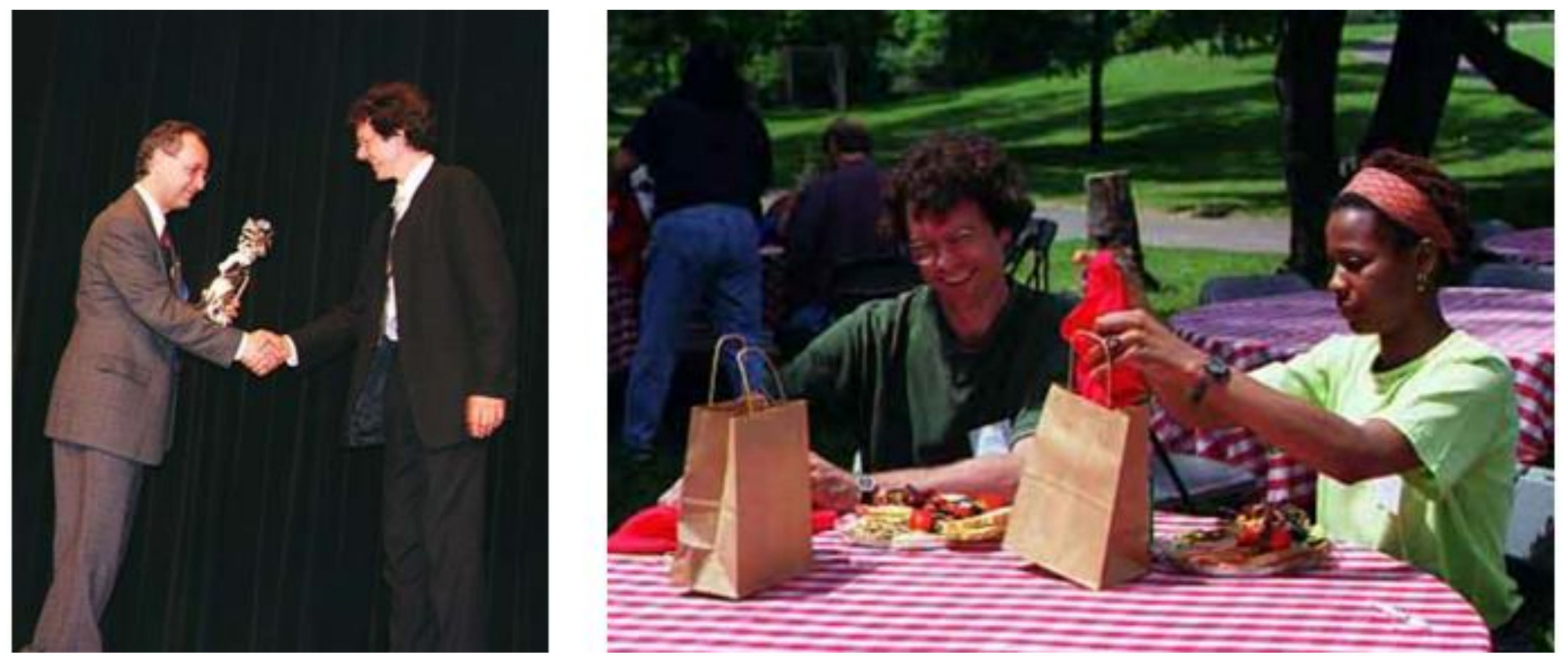
**Left**: GB presented Morten Meldal with a congratulatory bottle of champagne during the 16^th^ American Peptide Symposium closing banquet on 1 July 1999, in Minneapolis. **Right**: Much more casual: the end of the Symposium was celebrated on 2 July 1999 at a picnic for Barany alumni and friends, including MM with Phaedria Marie St. Hilaire.

In addition to all of the scientific proceedings, the Symposium included several social events in which Morten was an active participant^43^. These included a Speakers’ dinner at the Weisman Art Museum on the campus of the University of Minnesota, a final banquet/awards ceremony that featured the world premiere of the first (and likely only) opera ever composed about peptides (**Peptide Ångst***: La Triviata*) [194,195] and a reunion picnic on Friday, 2 July, for Barany Group alumni and special friends such as Morten. There was also a contest in the program book to convert GRANT to MONEY, and Morten’s solution, joint with St. Hilaire [GRANT → Grand → Brand → Brans → Brats → Boats → Boots → Books → Bonks → Bones → Boney → MONEY] earned him a champagne bottle, which was hand-delivered by GB at the banquet (Figure 9).

## 14. Summary and Conclusions

The hero (MM) of this perspective, and its senior author (GB), are (within experimental error) the same age, and have pursued both overlapping and orthogonal research interests over the course of their careers. While they may have occasionally competed, this was always friendly and with the utmost mutual respect and commitment to innovation and truth.

Our narrative here began with a selective and personal account of an independent period, starting in the mid-1970s and continuing through the 1980s, when conceptual development and experimental work conducted in New York City and Minneapolis led to an overhaul of solid-phase peptide synthesis strategies and tactics. There followed an extraordinarily fertile decade, the 1990s, when research programs in Minneapolis and in Copenhagen reinforced each other through exchanges of ideas and personnel, while opening up new areas.

Thus, the title *Copenhagen–Minneapolis axis* provided several versatile, robust PEG-based supports not only for peptide synthesis, but also for solid-phase organic synthesis (SPOS) and combinatorial chemistry—these materials could be applied in both batchwise and flow-through modes, and were compatible with biological assays carried out in aqueous environments. Further areas of convergent study led to advances in glycopeptide and PNA synthesis, among others. With the advent of mild, orthogonal protection schemes, the reliable preparation of acid-labile peptides of biological interest, including those with a myriad of post-translational modifications, was finally a reality. 

With the ushering in of a new millennium, Morten Meldal was exceptionally well positioned to achieve, together with his graduate student Christian Tornøe, the breakthrough SPOS experiments establishing the CuACC reaction, independently popularized by fellow Nobelist Sharpless as “click chemistry”. Importantly, this critical work was carried out on PEGA, the resin support developed less than a decade earlier in the Carlsberg Laboratory.

At the same time, the trajectory followed by Meldal and his team continued with even more variations on “click” chemistry, e.g., “electrophilic aromatic substitution cyclization–intramolecular click-cascade” (EASCy-ICC), and gave rise to new approaches in nanotechnology along with a host of novel functional molecules with interesting properties [196,197], all masterfully reviewed in Meldal´s Hirschmann award address [5]. In a particularly creative line of research, solid-phase phosphine- and carbene-based “green” catalysts, termed “organozymes”, were developed and applied [198,199]. Complementing Meldal’s academic résumé, his entrepreneurial side was expressed with the co-founding of three startup companies: Combio A/S (2000), Versamatrix A/S (2002), and Betamab APS (2019) [1].

The interests and foci of GB’s Minneapolis research program evolved as well, encompassing contributions to SPOS; protein folding and design; synthetic and mechanistic organosulfur chemistry; and the development of new approaches to teaching an organic chemistry laboratory course at the advanced undergraduate level^44^.

This perspective began by noting that Meldal first reported the CuACC method at an American Peptide Symposium, specifically the 17^th^ edition (2001) in San Diego. The day after the aforementioned meeting adjourned, a sizeable subset of its participants stayed an extra day for a satellite symposium entitled ”Crossroads of Chemistry and Biology”, co-organized by GB and Art Felix, on the occasion of Bruce Merrifield’s 80^th^ birthday [200]. Meldal’s 2022 prize is the first Nobel awarded to a peptide chemist since Merrifield was so honored in 1984, and we congratulate and applaud him and his achievements.

## Data Availability

Not applicable.

## Footnotes

1 By necessity, the literature cited herein cannot be comprehensive, and is skewed considerably to refereed papers reported out of Copenhagen (Meldal) and Minneapolis (Barany). For more details on the backgrounds and prior investigations—including preliminary results communicated at scientific meetings—in areas of interest to us, please refer to our original publications and review articles, in which we have tried to be conscientious and comprehensive with respect to proper attributions to precedents and/or independently pursued similar lines of inquiry.

2 “An *orthogonal* system is defined as a set of completely independent classes of protecting groups. In a system of this kind, each class of groups can be removed in any order and in the presence of all other classes” (verbatim footnote 4 from ref. [9]).

3 GB’s mentor, R. B. Merrifield, used the word “orthogonal” in a lecture at the 5^th^ American Peptide Symposium in 1977 in San Diego; a “note added in proof” to our proceedings report [10] states “During the discussion period for this paper we learned that the term ‘orthogonal’ has been used in a similar way in the laboratory of Dr. D. S. Kemp”. GB recalls being electrified by Dan Kemp’s lecture at the 4^th^ American Peptide Symposium in 1975 in New York City, but does not believe the word came up then, nor does it appear anywhere in Kemp’s proceedings report [11].

4 The anecdote that follows was recently confirmed by GB’s long-time friend and scientific confidante, Jed Fisher, who was in the conference room at the same time.

5 Henrik Franzyk worked with Morten Meldal from 1994–1996 and they published several important papers together [31,32,33].

6 While carrying out his first-ever SPPS in 1971, as part of the 6-week independent project phase of the legendary Rockefeller University summer biochemistry course, GB had the privilege to be informally mentored by Bernd Gutte, who was then wrapping up his postdoctoral studies. The resultant peptide proved to bind adenosine triphosphate (ATP), as reported at the 1972 Cold Spring Harbor Symposium on Muscle Contraction [36].

7 In a recent search (accessed 26 October 2023) of the Web of Science database (https://www.webofscience.com/), the word “orthogonal” appeared over 3400 times in the *titles* of chemistry articles.

8 Chemical entities that react specifically with thiol, amino, or imidazole side-chains in proteins under aqueous conditions have been studied extensively since the 1960s, as has been reviewed frequently [87,88].

9 The Dts group is one of just a handful of protecting groups that cover *both* free valences of nitrogen on aliphatic or aromatic amines [9,90,91,92,93].

10 Because this compound (**1**) is formally a thioanhydride of *O*-ethyl xanthate (the product of ethanol and carbon disulfide), we refer to it colloquially as “ethyl xanthic anhydride” (EXA).

11 An alternative to the ZWK reaction starts with 1,2,4-dithiazolidine-3,5-dione (DtsNH) [89,95], which reacts with alkyl halides in the presence of base to provide Dts-amines. This Dts variation of the classical Gabriel synthesis was elegantly pioneered by Wood and coworkers [96], although it should be noted that GB pursued such approaches (never published) while still at The Rockefeller University and later with undergraduates carrying out directed studies at the University of Minnesota.

12 In 2005, we finally achieved [97] a long-elusive goal [9] to introduce the Dts group in a *single step* by reaction of bis(chlorocarbonyl)disulfane [Cl(C=O)SS(C=O)Cl] [9,98,99] with bis(trimethylsilyl)amines [TMS_2_NR]. For this method, the TMS group acts as a “large proton”, in the sense that two equiv of TMSCl are formed as co-products, rather than HCl (or HCl salts, if the reactions are carried out in the presence of base).

13 *Point of personal privilege*: The first author (MJB) of our 2005 *J. Am. Chem. Soc*. communication [97], currently an eminent historian of mathematics at the University of Edinburgh in Scotland, was at the time a Minnesota high school student competing in the nationwide Intel Science Talent Search. The two enabling ideas, namely the use of Cl(C=O)SS(C=O)Cl and the use of TMS_2_NR, date back respectively to GB’s graduate studies with RBM in the mid-1970s and to RPH’s graduate studies with GB in the late 1980s. The Nobel laureate coauthor (RBM) enthusiastically signed off on the manuscript, which turned out to be the final one of his illustrious career.

14 To the best of our knowledge, the first examples for conversion of R’O(C=S)NHR to their thermodynamically more stable R’S(C=O)NHR isomers were discovered toward the end of the 19^th^ century. Experienced organosulfur chemists are well familiar with this transformation, which can occur spontaneously, or upon heating, or with catalysis, all depending on the precise nature of the substrate. In the "name game" of organic chemistry, the rearrangement is variously associated with Alexander Schönberg, or Melvin Newman with Howard Kwart, or (tangentially) Derek Barton with Stewart McCombie.

15 Specifically, “when you are on the ground, pick something up”.

16 Aqueous hydroxide under ambient conditions removes primary and secondary alkyl esters, although not tertiary alkyl esters—these latter require TFA (or moderate acids of comparable strength) under mostly anhydrous conditions (sometimes, a relatively small amount of water is added as a carbocation scavenger).

17 An anecdote from the 1970s might be instructive to young scientists nowadays. As a graduate student learning about peptide synthesis, GB admired the work of the Merck group on the “controlled polymerization” of *N*-carboxyanhydrides (NCAs) [104,105] for stepwise synthesis of small peptide fragments. The NCA approach is uniquely problematic with respect to the amino acid glycine (equilibration with its isocyanate isomer), so the sulfur analogue *N*-thiocarboxyanhydride (NTA) has to be applied to incorporate this residue [105,106]. Just as the Dts group is established in one more step beyond creating an Etc-amine derivative, so too are NTAs. Upon re-reading the classic review by Zumach and Kühle [89] in which he had first encountered a drawing of the lead heterocycle that he was to later reconfigure to Dts, GB realized that it was inevitable that the Merck group would *also* be aware of that *same* heterocyle—this realization resulted in several years of low-level anxiety and worrying about being “scooped”. GB’s preliminary Dts work was presented for the very first time in 1975 as a poster at the 4^th^ American Peptide Symposium in New York City [a crosstown bus ride away from the Rockefeller campus]. Dan Veber of Merck visited the poster, and was gracious with his praise. As an aside, he remarked, “…you know, we actually made Dts-Gly-OEt and had it sitting in a vial in the laboratory, but were unable to proceed further because nobody could figure out how to remove the ester without destroying the heterocycle” (paraphrased, not exact quote). GB concluded that certain scientific problems succumb only to a single-minded focus, and that even outstanding and experienced scientists can miss something that is entirely obvious in hindsight.

18 Later, when Dts-amino acid derivatives were needed for SPPS (next section of this perspective), the free acids were released *in situ* from their DCHA salts by brief but vigorous agitation of the latter, dissolved or suspended in CH_2_Cl_2_, with Dowex 50 (hydrogen form) ion-exchange resin, followed by filtration.

19 As a counterpoint that directly ties in with our concept of orthogonality, Dts-amines are *unusually stable* under acidic conditions. We believe that this is due to the planarity of the heterocyclic ring, which allows for electron delocalization (evidenced by shorter bond lengths), and imparts a certain level of aromatic stabilization.

20 Use of trialkyl- and triarylphosphines [109,110,111,112] in the presence of water is a well-known method to reduce disulfides to the corresponding thiols. When we carried out the obvious control experiment under anhydrous conditions, we discovered that Dts-amines were converted to the corresponding isocyanates, along with COS and the appropriate phosphine sulfide. This observation led directly to novel and highly efficient procedures for the generation of phosphorothioates using first 1,2,4-dithiazolidine-3,5-dione (DtsNH) and then later 3-ethoxy-1,2,4-dithiazoline-5-one (EDITH) [113,114].

21 A somewhat surprising way to achieve Dts removal is under the acidic reductive conditions (pH 5.2) of color development in a standard amino acid analyzer. Specifically, the hydrindantin removes Dts, at least partially, and the ninhydrin reacts with the released amines to provide the purple chromophore (570 nm) that allows detection and quantification of the parent compounds [43,46].

22 Application of MAc exemplifies a more “potent” thiol balanced by NMM, which is a weaker base. Interestingly, MAc is a “high κ” thiol, insofar as Dts cleavage is rate-limiting and no carbamoyl disulfide intermediate was ever isolated (see Scheme 4 as well as ref. [43]). In the more polar solvent DMF, no base is needed, and in fact the reaction is rapid in the presence of HOBt [which is a *weak acid*; it is included in the deprotection cocktail so as to neutralize any trace of amine impurities in DMF].

23 The adjective “difficult” gained traction in the 1980s through the work of Charles Deber, Stephen Kent, and Bob Sheppard [119,120,121,122,123,124], who documented that some couplings during stepwise SPPS, regardless of protecting group (Boc, Fmoc, or Dts), were reproducibly slow and/or did not go to completion despite lengthier coupling times, higher temperatures, microwave irradiation, more reactive activation methods, and/or addition of chaotropic agents to the coupling milieus. The consensus view, developed by the aforementioned pioneers and still held today, is that these couplings are difficult due to the formation of secondary structure (aggregation) in the resin-bound peptides.

24 The preceding paragraph used the “Achilles heel” metaphor from Greek mythology. In a different vein, GB recalls being admonished by his mother with words to the effect “Dts may have started out as your brainchild, but it has now grown into an unruly teenager!”

25 This approach accepts a modest level of additional crosslinking, readily quantified, whenever a bifunctional diacid is coupled on *both* sides to the resin-bound amines. Typically, about two-thirds of the PEG chains retain an accessible end group that can be modified further to eventually initiate SPPS or SPOS. The ultimate properties of such statistically hetero-diverse PEG–PS do not appear to be adversely affected by the added crosslinking.

26 When the starting PS component of commercial PEG–PS is MBHA–PS, the resin material is labile to anhydrous HF, and therefore not suitable for stepwise SPPS by Boc chemistry. However, PEG–PS is easily reformulated starting from aminomethyl–PS, whereupon it is entirely suitable for Boc chemistry with a final HF cleavage step.

27 The Twins were on their way to their second World Series championship in four years. A box score for this particular game can be found at https://www.baseball-reference.com/boxes/MIN/MIN199110090.shtml.

28 GB’s opening joke was a tongue-in-cheek apology for appearing tired, on account of not being accustomed to staying up so late.

29 See: https://www.facebook.com/abeautifulequation/photos/a.632898940054321/5368004756543692/ For more about Bohr, see: https://www.nobelprize.org/prizes/physics/1922/bohr/biographical/ To celebrate the 100^th^ anniversary of the Bohr model of the atom, GB collaborated with Alex Vratsanos on “Great Dane”, a crossword puzzle published in the 20 September 2013 issue of *The Chronicle of Higher Education*, as reproduced and discussed at: http://www1.chem.umn.edu/groups/baranygp/puzzles/greatdane/.

30 Live broadcast of a Joe Biden-led committee hearing of the United States Senate concerning confirmation of Clarence Thomas in light of explosive allegations of sexual harassment from Professor Anita Hill.

31 In the section that follows, as well as a later section entitled “Invention of Further PEG-based Supports …”, we highlight novel support materials (PEGA, POEPS, POEPOP, POEPS-3, and SPOCC) invented by Meldal and coworkers. No attempt has been made to name the monomers leading to these materials in a manner entirely consistent with how we have described the chemistry leading to various generations of PEG–PS and CLEAR supports.

32 Shortly after PRH presented preliminary findings from Minneapolis in 1994 at the 23^rd^ European Peptide Symposium in Braga, Portugal, Morten Meldal let us know that work along the same lines was already underway in Copenhagen and had been presented seven weeks earlier at the XVII^th^ International Carbohydrate Symposium in Ottawa, Canada. In a cordial conversation, GB suggested that the first full paper on this topic should be submitted by the Copenhagen group, followed soon thereafter by a paper from Minneapolis.

33 The auxiliary nucleophile forms a yellow ion-pair with unreacted amino groups on the resin, thereby allowing visual monitoring of the progress of the reaction. In addition, the active species for coupling is likely to be the more reactive Dhbt ester, formed *in situ* by transesterification that displaces the pentafluorophenyl (Pfp) ester.

34 In keeping with the title of this perspective, we have already described innovative work in the field of glycopeptide synthesis where there was some synergy or overlap from the efforts in Copenhagen and Minneapolis. Meldal’s many additional contributions and successes in the field fall outside our scope, but the interested reader can easily find them summarized elsewhere [148,149]. Methodologies developed at Carlsberg were readily extended to combinatorial libraries, for example in a paper by St. Hilaire et al. [150] which described carboxylic acid-encoded glycopeptide libraries.

35 This perspective will not delve into Knud Jensen’s highly creative independent idea of *carbopeptides*, which he brought with him to Minneapolis and devoted considerable time and effort to achieving proof-of-concept results. The goal was to use a sugar (specifically, d-galactopyranose) as a template to start four identical peptide strands (initially, six amino acid residues each, plus β-alanine “spacers”) that might interact with each other to achieve stable secondary or tertiary structures as protein models (4-helix bundles) or multiple antigenic peptides (MAPs). Because the expertise and resources of his laboratory facilitated progress towards this ambitious goal, GB was delighted to coauthor the first report in 2000 in the *J. Pept. Sci.* [151] but he then encouraged KJJ to elaborate on this work on his own to launch his independent academic career in the Department of Chemistry at the University of Copenhagen. Follow-up papers from Jensen and coworkers ultimately came out in the first few years of the 21^st^ century [152,153,154,155].

36 CLEAR supports are explained in a later section of this perspective.

37 Sweden and Denmark are close enough that people headed to the south of Sweden often fly into the Copenhagen airport, and then take a train or automobile across the Øresund Bridge. In the framing of this perspective’s title, one might say that Maria Kempe was an “honorary” member of Copenhagen’s scientific circle.

38 We may write about the “Barcelona–Minneapolis connection” at a different time for a different venue. For now, we interpret the title “Copenhagen–Minneapolis axis” of this perspective generously, so as to include Fernando Albericio’s important work on ChemMatrix.

39 This section deviates somewhat from our overall framing for this perspective, since to the best of our knowledge, Morten Meldal’s research programs never tackled PNA. Nonetheless, we include it because PNA originated from Copenhagen, and Meldal-trained Knud Jensen was one of the leaders of the Minneapolis effort in this area. Our discussion here is focused *only* on Dts chemistry; separate efforts from Minneapolis using Fmoc chemistry have been communicated elsewhere [180].

40 If the end goal for the synthetic PNA is hybridization to target genomic DNA for mutational analysis, diagnostics, and myriad additional applications, then PNA that is assembled by a segment approach is far less likely to lead to false positive results, which would otherwise be a distinct risk whenever there are single-deletion “failure” sequences due to stepwise synthesis. In collaboration with Francis Barany and Robert Hammer, we began discussing these concepts in 1993; there is a straight line from those discussions to our invention of “universal zip-code arrays” which were first filed to the United States Patent and Trademark Office (USPTO) in 1997 and disclosed in a 2001 *Journal of Molecular Biology* paper [188].

41 The previous footnote mentions a PNA segment approach. We began working toward this goal around 1996 under a grant from the National Institute of Science and Technology (NIST), in collaboration with Robert Hammer, who had graduated from our Minneapolis laboratory in 1990 and was by then an independent Assistant Professor at Louisiana State University (LSU) in Baton Rouge. In never-published work, Bob’s LSU postdoctoral fellow Kristofer Moffett used chemistry with three degrees of orthogonality [*N*^ω^-Fmoc/side-chain Z/*C*^ω^-allyl ester] to synthesize all 16 suitably protected Fmoc-PNA dimers, and made some progress towards combining these to create a few PNA tetramers. Since then, there have been several literature reports on short PNA oligomers [189,190,191].

42 The two co-Chairs had been colleagues for several years at the University of Minnesota, respectively in the Department of Chemistry in the Institute of Technology (since renamed College of Science and Engineering) and the Department of Laboratory Medicine and Pathology in the Medical School. About a year before the 16^th^ American Peptide Symposium, GBF moved to Florida Atlantic University.

43 A full photo gallery from the meeting can be found at http://www1.chem.umn.edu/16aps/Photoindex.html.

44 These various activities are outside of the scope of the present perspective, but may be the subject of future writings.

## Abbreviations

A, adenine or the corresponding PNA building block; AA, amino acid residue [free or protected, depending on context, and l-configuration unless noted otherwise]; ABz, 2-aminobenzamide; Ac, acetyl; Acm, acetamidomethyl; Ac_2_O, acetic anhydride; ACP, acyl carrier protein; AgOTf, silver trifluoromethanesulfonate; All, allyl; Aloc, allyloxycarbonyl; BAL, **b**ackbone **a**mide **l**inker; BF_3_**^.^**Et_2_O, boron trifluoride diethyl etherate; Boc, *tert*-butyloxycarbonyl; BSA, *N*,*O*-bis(trimethylsilyl)acetamide); BSU, *N*,*N*′-bis(trimethylsilyl)urea; C, cytosine or the corresponding PNA building block; CD, circular dichroism; cHex, cyclohexyl; CLEAR, **C**ross-**L**inked **E**thoxylate **A**crylate **R**esin; ClZ, 2-chlorobenzyloxycarbonyl; CuACC, copper(I)-catalyzed azide–alkyne cycloaddition(s); DCC, *N*,*N*′-dicyclohexylcarbodiimide; DCHA, dicyclohexylammonium (salt); DEAE, diethylaminoethyl (for ion-exchange chromatography); Dhbt-OH, 3-hydroxy-1,2,3-benzotriazin-4(3*H*)-one; DIEA, *N*,*N*-diisopropylethylamine; DIPCDI, *N*,*N*′-diisopropylcarbodiimide; DMA, *N*,*N*-dimethylacetamide; Dmaetc, 2-(dimethylamino)ethoxythiocarbonyl; DMAP, 4-dimethylaminopyridine; DMF, *N*,*N*-dimethylformamide; Dts, **d**i**t**hia**s**uccinoyl; DTT, (2*S*,3*S*)-1,4-dimercaptobutane-2,3-diol; EDCI, 1-ethyl-3-(3-dimethylaminopropyl)carbodiimide hydrochloride; EDITH, 3-ethoxy-1,2,4-dithiazoline-5-one; EDT, ethane-1,2-dithiol ≡ HSCH_2_CH_2_SH; Emc, (ethylthio)carbonyl; Eoc, ethoxycarbonyl; ESI-MS, electrospray ionization mass spectrometry; Et, ethyl; Etc, ethoxythiocarbonyl; EXA, ethyl xanthic anhydride; Fmoc, 9-fluorenylmethyloxycarbonyl; Fmoc-OSu, (*N*-(9-fluorenylmethyloxycarbonyloxy)succinimide); G, guanine or the corresponding PNA building block; Glp, pyroglutamyl (residue); HBTU, *N*-[(1*H*-benzotriazol-1-yl)(dimethylamino)methylene]-*N*-methylmethanminium hexafluorophosphate *N*-oxide; HCTU: 2-(1*H*-6-chlorobenzotriazole-1-yl)-1,1,3,3-tetramethyluronium hexafluorophosphate; HF, (anhydrous) hydrogen fluoride; HMPA, 4-(hydroxymethyl)phenoxyacetic acid; HOAc, acetic acid; HOBt, 1*H*-1,2,3-benzotriazol-1-ol ≡ 1-hydroxybenzotriazole; iPrdtc, (*iso*-propyldithio)carbonyl; IRAA, “internal reference” amino acid; KHMDS, potassium bis(trimethylsilyl) amide; MAc, *N*-methylmercaptoacetamide; MAPs, multiple antigenic peptides; MBHA, 4-methylbenzhydrylamine (resin); Meb, 4-methylbenzyl; NaBH_4_, sodium borohydride; MPEG, (monofunctional) poly(ethylene glycol) methyl ether; NaCNBH_3_, sodium cyanoborohydride; NEM, *N*-ethylmorpholine; NMM, *N*-methylmorpholine; NMP, *N*-methyl-2-pyrrolidinone; NMR, nuclear magnetic resonance; ONb, *ortho*-nitrobenzyl (ester); OtBu, *tert*-butyloxy; PAB, *p*-alkoxybenzyl; PAL, tris(alkoxy)benzylamide ≡ peptide amide linker; PAM, phenylacetamidomethyl; Pbf, 2,2,4,6,7-pentamethyldihydrobenzofuran-5-sulfonyl (arginine protecting group); PDT, propane-1,3-dithiol ≡ HSCH_2_CH_2_CH_2_SH; PEG; poly(ethylene glycol); PEGA: poly(ethylene glycol) dimethylacrylamide (resin); PEG–PS, poly(ethylene glycol)–*graft*–polystyrene support; Pfp, pentaflurophenyl (ester); PNA, peptide nucleic acid; PS, polystyrene (resin), typically cross-linked with 1% divinylbenzene, and used as microporous beads; RANTES, **r**egulated on **a**ctivation, **n**ormal **T** cell **e**xpressed and **s**ecreted (a chemokine); RP-HPLC: **r**eversed-**p**hase **h**igh **p**erformance **l**iquid **c**hromatography; Snm, (*N*-methyl-*N*-phenylcarbamoyl)sulfenyl; SPOCC, **s**uper**p**ermeable **o**rganic **c**ombinatorial **c**hemistry (resin); SPOS, **s**olid-**p**hase **o**rganic **s**ynthesis; SPPS, **s**olid-**p**hase **p**eptide synthesis; SST, somatostatin; T, thymine or the corresponding PNA building block; tBu, *tert*-butyl; tBuOTf, *tert*-butyltrifluoroacetate; TFA, trifluoroacetic acid; TFE, trifluoroethanol; THF, tetrahydrofuran; TMS, trimethylsilyl; TMSOTf, trimethylsilyl trifluoromethanesulfonate; Trt, trityl ≡ triphenylmethyl; TsOH, *p*-toluenesulfonic acid; Tyr(NO_2_), 3-nitrotyrosine; UDP, uridine 5′-(α-d-galactopyranosyl dihydrogen diphosphate); Z, benzyloxycarbonyl; ZWK, Zumach–Weiss–Kühle (reaction to establish the Dts heterocycle).
